# Identification of Cell Type-Specific Differences in Erythropoietin Receptor Signaling in Primary Erythroid and Lung Cancer Cells

**DOI:** 10.1371/journal.pcbi.1005049

**Published:** 2016-08-05

**Authors:** Ruth Merkle, Bernhard Steiert, Florian Salopiata, Sofia Depner, Andreas Raue, Nao Iwamoto, Max Schelker, Helge Hass, Marvin Wäsch, Martin E. Böhm, Oliver Mücke, Daniel B. Lipka, Christoph Plass, Wolf D. Lehmann, Clemens Kreutz, Jens Timmer, Marcel Schilling, Ursula Klingmüller

**Affiliations:** 1 Division Systems Biology of Signal Transduction, German Cancer Research Center (DKFZ), INF 280, Heidelberg, Germany; 2 Translational Lung Research Center (TLRC), German Center for Lung Research (DZL), Heidelberg, Germany; 3 Institute of Physics, University of Freiburg, Germany & BIOSS Centre for Biological Signalling Studies, University of Freiburg, Germany; 4 Division Epigenomics and Cancer Risk Factors, German Cancer Research Center (DKFZ), INF 280, Heidelberg, Germany; 5 Regulation of Cellular Differentiation Group, Division Epigenomics and Cancer Risk Factors, German Cancer Research Center (DKFZ), INF 280, Heidelberg, Germany; Johns Hopkins University, UNITED STATES

## Abstract

Lung cancer, with its most prevalent form non-small-cell lung carcinoma (NSCLC), is one of the leading causes of cancer-related deaths worldwide, and is commonly treated with chemotherapeutic drugs such as cisplatin. Lung cancer patients frequently suffer from chemotherapy-induced anemia, which can be treated with erythropoietin (EPO). However, studies have indicated that EPO not only promotes erythropoiesis in hematopoietic cells, but may also enhance survival of NSCLC cells. Here, we verified that the NSCLC cell line H838 expresses functional erythropoietin receptors (EPOR) and that treatment with EPO reduces cisplatin-induced apoptosis. To pinpoint differences in EPO-induced survival signaling in erythroid progenitor cells (CFU-E, colony forming unit-erythroid) and H838 cells, we combined mathematical modeling with a method for feature selection, the L_1_ regularization. Utilizing an example model and simulated data, we demonstrated that this approach enables the accurate identification and quantification of cell type-specific parameters. We applied our strategy to quantitative time-resolved data of EPO-induced JAK/STAT signaling generated by quantitative immunoblotting, mass spectrometry and quantitative real-time PCR (qRT-PCR) in CFU-E and H838 cells as well as H838 cells overexpressing human EPOR (H838-HA-hEPOR). The established parsimonious mathematical model was able to simultaneously describe the data sets of CFU-E, H838 and H838-HA-hEPOR cells. Seven cell type-specific parameters were identified that included for example parameters for nuclear translocation of STAT5 and target gene induction. Cell type-specific differences in target gene induction were experimentally validated by qRT-PCR experiments. The systematic identification of pathway differences and sensitivities of EPOR signaling in CFU-E and H838 cells revealed potential targets for intervention to selectively inhibit EPO-induced signaling in the tumor cells but leave the responses in erythroid progenitor cells unaffected. Thus, the proposed modeling strategy can be employed as a general procedure to identify cell type-specific parameters and to recommend treatment strategies for the selective targeting of specific cell types.

## Author Summary

A major challenge in the development of therapeutic interventions is the selective inhibition of a signal transduction pathway in one cell type such as a cancer cell leaving the other cell type such as a healthy cell as unaffected as possible. Here, we propose a new approach that combines mathematical modeling based on quantitative experimental data with statistical methods. We demonstrate based on simulated data that our approach can determine which parameters are the same and which parameters differ in two exemplary cell types. We compare a lung cancer cell line to the precursor cells of red blood cells. We show that the same signal transduction network induced by erythropoietin (EPO), a hormone that is frequently employed to treat anemia in cancer patients, regulates survival of both cell types. Based on our experimental data in combination with our computational approach, we identify seven cell type-specific differences in this signaling pathway. Our strategy allows predicting therapeutic targets that could be inhibited to interfere with survival of lung cancer cells while leaving production of red blood cells unaffected.

## Introduction

Lung carcinoma is one of the leading causes of cancer-related deaths worldwide. The main types of lung cancer are small-cell lung carcinoma (SCLC) and non-small-cell lung carcinoma (NSCLC). NSCLC is the most frequent form with a prevalence of around 85% and can be classified in squamous-cell carcinoma, large-cell carcinoma, and adenocarcinoma which is the most prevalent subgroup (40%) [1]. Because lung cancer metastasizes already at early stages independent of the tumor size [2], most of the patients receive chemotherapeutic agents such as cisplatin. As a side effect of chemotherapy, as well as due to tumor-related effects, anemia frequently occurs [3]. Anemia is treated either by blood transfusion or by erythropoiesis stimulating agents (ESAs) such as erythropoietin (EPO) alfa or beta [4]. EPO is the key regulator of red blood cell production and ensures survival, proliferation and differentiation of erythroid progenitors at the colony forming unit-erythroid (CFU-E) stage in the fetal liver, the adult bone marrow and spleen. Biosynthesis of EPO in the kidney is stimulated by reduced blood oxygen levels [5]. Unfortunately, recent studies suggested that EPO treatment could reduce the overall survival of NSCLC patients [6]. Furthermore, expression of the EPO receptor (EPOR) has been detected in some tumors and cancer cells including NSCLC cells [7–10]. Co-expression of EPO and the EPOR has been shown to be associated with poor survival of NSCLC patients, even at stage I [11]. Because of these ambivalent effects, the treatment of lung cancer patients with EPO in the context of cancer-related anemia is controversially discussed [12, 13]. In addition to clinically administered recombinant EPO, endogenous EPO produced in the kidney may also influence cancer cells.

It has been speculated that EPO might affect the survival of cancer cells [14]. The key signaling pathway that is activated by EPO binding to the EPOR and that is involved in survival signaling is the Janus kinase (JAK)2 / signal transducer and activator of transcription (STAT)5 pathway. EPOR is a member of the cytokine receptor superfamily and is present at the cell surface as a homodimer [15]. Upon EPO binding, the receptor undergoes conformational changes and activates the pre-bound tyrosine kinase JAK2 [16]. The activated JAK2 is transphosphorylated and phosphorylates (p) tyrosine residues on the EPOR cytoplasmic domain that serve as docking site for the interaction with Src-homology (SH)2-domain containing proteins. The phosphorylated EPOR-JAK2 complex is able to bind and phosphorylate the latent transcription factor STAT5. Subsequently, pSTAT5 forms dimers that translocate to the nucleus, where they induce the transcription of target genes. JAK2/STAT5 target genes such as cytokine-inducible SH2-containing protein (*CISH* mRNA, translated to CIS) and suppressor of cytokine signaling 3 (*SOCS3* mRNA, translated to SOCS3) are expressed that act as negative regulators of JAK2/STAT5 signaling at the EPOR/JAK2 level [17]. Specifically, CIS inhibits STAT5 activation by binding to the EPOR, whereas SOCS3 binds to the activated receptor and the kinase domain of JAK2, thereby inhibiting its tyrosine kinase activity [18–21]. In addition, protein tyrosine phosphatases (PTPs) negatively regulate the activated EPOR-JAK2 complex [21]. It has been shown that the PTP SHP1 negatively regulates EPOR signaling in hematopoietic cells [22].

EPO acts on the erythroid lineage, particularly on CFU-E cells residing in the bone marrow of human adults. It is difficult to obtain primary human CFU-E cells in adequate numbers to perform time-resolved biochemical experiments required for mathematical modeling. However, murine CFU-E cells can be readily purified from fetal mouse liver preparations subjected to negative depletion with lineage-specific antibodies [23] and thereby can be isolated in sufficient quantities for data-based mathematical modeling. Therefore, we employed murine CFU-E cells as a proxy for human CFU-E cells and as representative for healthy erythroid cells.

Recently, an ordinary differential equation (ODE) model of the EPO-induced JAK2/STAT5 pathway in murine CFU-E cells was reported [19] that focused on the distinct roles of SOCS3 and CIS and linked the integrated nuclear pSTAT5 response to cell survival. To investigate differences of EPO-induced JAK2/STAT5 signaling in hematopoietic and non-hematopoietic cell types like cancer cells, such an ODE model can be generalized. The parameters of the generalized mathematical model can be estimated individually using time- and dose-resolved quantitative data for the studied cell types. A major challenge is to identify significant differences in parameter values that are characteristic of a respective cell type. In general, one could discriminate between cell type-specific parameters and cell type-independent parameters. Mathematically, all combinations of parameters being either cell type-specific or cell type-independent have to be tested. Finding the exact solution of this model selection task is challenging as the number of candidate models grows exponentially with the number of model parameters [24]. Established approaches to approximate the solution of such a selection task have been developed mainly in classical statistics and comprise approaches such as lasso (least absolute shrinkage and selection operator) [25], elastic net [26], forward selection and backward elimination [27], and combinations thereof [28]. These approaches favor small models by penalizing increasing numbers of parameters. Their main difference is the metric quantifying the model complexity. In the lasso approach, the L_1_ metric, i.e., the absolute value of differences to zero is used. It has been shown that under certain conditions, the L_1_ metric produces similar results as the L_0_ metric that penalizes only the number of parameters [29]. However, due to convexity and continuity of the L_1_ metric, it is considered favorable over L_0_ [30]. The L_2_ metric, on the other hand, does not lead to a sparse (parsimonious) model and is therefore not suitable for our task. Because L_1_ regularization techniques have been mainly developed for linear models, only a subset of existing algorithms can be employed for models that are nonlinear, which is a prevalent property of mathematical models based on coupled ODEs. Additional issues arising from nonlinearity comprise local optima, non-identifiabilities, and decreased performance of numerical calculations in general. To tackle these challenges, state-of-the-art implementations for optimization in nonlinear ODE systems have been developed [31]. We utilize these established approaches and extend their functionality by identifying parameter differences with L_1_ regularization. For a successful implementation, strategies to consider discontinuities in the derivatives and adaptation of convergence criteria have to be taken into account [32].

Here, we show on the basis of simulated data that our implementation for modeling of nonlinear ODE systems in combination with an L_1_ approach is a suitable method to infer cell type-specific parameters of two cell types and demonstrate the interpretation of the results. This approach is then applied to assess parameter differences in EPO-induced JAK2/STAT5 signaling between the NSCLC cell line H838 that expresses the EPOR [9, 10, 33] and CFU-E cells. Furthermore, a sensitivity analysis of the biological readout is performed to discover potential targets for intervention that specifically reduce the effects of EPO on the cancer cells while leaving the EPO-induced survival of hematopoietic cells unaffected.

## Results

### Expression and function of EPOR in the NSCLC cell line H838

**Fig 1:**
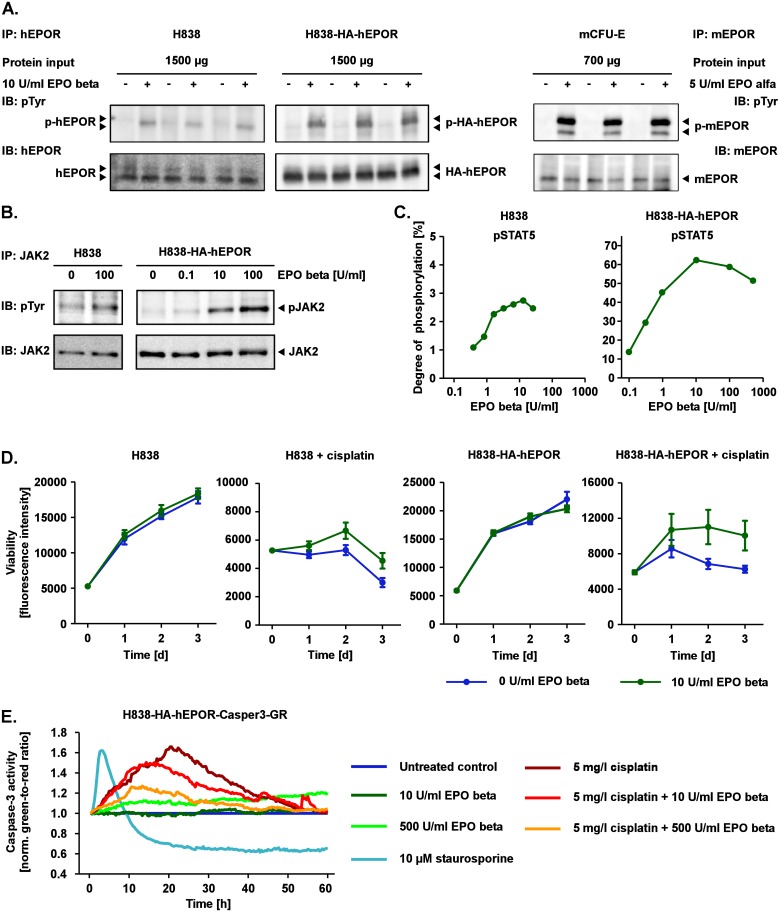
Functional and signaling-competent EPOR in the NSCLC cell line H838 and CFU-E cells. (A) Left panel: H838 and H838 cells stably overexpressing HA-tagged human EPOR (H838-HA-hEPOR) were either left untreated (-) or stimulated with 10 U/ml EPO beta (+). Cells were lysed after 10 min and hEPOR proteins were subjected to immunoprecipitation (IP, MAB 307, R&D) and phosphorylated (p) EPOR (4G10, Merck Millipore) and total EPOR (C-20, Santa Cruz) were detected by quantitative immunoblotting (IB). The complete immunoblot is shown in S1 Fig. Right panel: murine (m) CFU-E cells were either left untreated (-) or stimulated with 5 U/ml Epo alfa (+). Cells were lysed after 10 min and mEPOR proteins were subjected to IP (M-20, Santa Cruz). pEPOR (4G10, Merck Millipore) and total EPOR (M-20, Santa Cruz) were detected by IB with chemiluminescence by CCD camera. The experiment was performed in biological triplicates. (B) H838 and H838-HA-hEPOR cells were treated with the indicated doses of EPO beta. Cells were lysed after 10 min and JAK2 proteins were subjected to IP. pJAK2 and total JAK2 were detected by IB with chemiluminescence by CCD camera. (C) H838 and H838-HA-hEPOR cells were treated with the indicated doses of EPO beta. Cells were lysed after 20 min and STAT5 proteins were subjected to IP. The degree of phosphorylation of STAT5 was measured with mass spectrometry. For H838 cells, the average degree of STAT5 phosphorylation based on biological duplicates is shown. (D) H838 and H838-HA-hEPOR cells were treated for three days with 5 mg/l cisplatin or left untreated. Additionally, cells were treated with 10 U/ml EPO beta and the cell viability was measured with CellTiter-Blue assay. The error bars represent standard deviation of biological replicates (n ≥ 5). The experiment was performed on two independent days (second replicate in S4 Fig). (E) H838-HA-hEPOR cells expressing the Casper3-GR FRET-based sensor (H838-HA-hEPOR-Casper3-GR) were treated with 5 mg/l cisplatin, EPO beta, a combination of both or left untreated. Staurosporine (10 μM) was used as positive control for induction of apoptosis. Casper3-GR FRET signal was measured by life-cell imaging for 60 hours. Caspase-3 activity was determined based on the green-to-red ratio and normalized to the untreated control (n = 2, second replicate in S5 Fig).

We previously showed that the NSCLC cell line H838 expresses the EPOR at the mRNA and protein level, albeit at rather low levels [10]. To more robustly detect EPO-induced phosphorylation of signaling components and expression of target genes in this cell line, we stably expressed high levels of HA-tagged human EPOR (HA-hEPOR) in H838 cells by retroviral transduction (H838-HA-hEPOR). The amount of EPOR protein present in the human lung cancer cell line H838 and its derivative H838-HA-hEPOR was determined by immunoprecipitation followed by quantitative immunoblotting and compared to the amount present in mouse CFU-E cells (Fig 1A). A protein band in the immunoblot that corresponds to the EPOR was detected in the analyzed cell types. As shown in S1A and S1B Fig, the hEPOR levels in H838-HA-hEPOR cells were increased 170-fold compared to H838 cells. The shift in molecular weight of the EPOR in H838-HA-hEPOR cells is most likely due to the HA-tag introduced into the EPOR. For absolute quantification of receptor expression levels, signal intensities of purified protein standards of mouse or human GST-tagged EPOR [34] were used to establish calibration curves and to subsequently estimate the number of EPOR molecules per cell (S1D Fig). As shown in S1 Table, H838-HA-hEPOR cells harbor 620 000 ± 200 000 hEPOR molecules per cell, H838 cells 3 600 ± 1 200 hEPOR molecules per cell and CFU-E cells 4 300 ± 2 200 mEPOR molecules per cell.

To assess the activation of signal transduction we examined ligand-induced phosphorylation of the EPOR (Fig 1A). H838 and H838-HA-hEPOR cells were treated for 10 min with 10 U/ml EPO beta and CFU-E cells for 10 min with 5 U/ml EPO alfa or were left untreated. In each case, a band in the immunoblot corresponding to the phosphorylated EPOR was detectable in the treated samples, but was absent in the unstimulated controls. The amount of pEPOR in H838-HA-hEPOR upon stimulation with 10 U/ml EPO beta was only approximately 12-fold increased compared to H838 (S1C Fig), indicating that in cells expressing elevated levels of the EPOR such as H838-HA-hEPOR possibly the transport of the receptor to the plasma membrane or phosphorylation of the EPOR by JAK2 might be limiting. S2A and S2B Fig show that EPO alfa and EPO beta activate EPO-induced signaling at a comparable extent.

Further, we examined EPO-induced JAK2/STAT5 survival signaling in the NSCLC cell line H838 and its derivative H838-HA-hEPOR because it had been reported that EPO induces survival signaling via the JAK2/STAT5 pathway in CFU-E cells [19]. In H838 cells, pJAK2 was detected upon stimulation with 100 U/ml and in H838-HA-hEPOR an EPO dose-dependent increase in JAK2 phosphorylation was observed (Fig 1B). Because the latent transcription factor STAT5 is a key mediator of the JAK2/STAT5 signaling pathway, we determined the degree of STAT5 phosphorylation by mass spectrometry in H838 and H838-HA-hEPOR cells treated with different doses of EPO beta (Fig 1C). The highest degree of STAT5 phosphorylation was observed at 10 U/ml EPO beta, with approximately 60% of STAT5 in H838-HA-hEPOR cells being phosphorylated and 3% in H838 cells. As quantified in S2C and S2D Fig and summarized in S1 Table both H838 and H838-HA-hEPOR harbor approximately 1 200 JAK2 and 90 000 STAT5 molecules compared to 24 000 JAK2 and 20 000 STAT5 molecules in CFU-E. To also consider the cytoplasmic and nuclear volume of the cell types analyzed, trypsinized cells were analyzed by fluorescence microscopy and the diameter of the cell and of the nucleus was determined (S3A and S3B Fig). CFU-E cells with a cell volume of 700 μm^3^ and a nuclear volume of 300 μm^3^ are much smaller compared to H838 cells that have a cell volume of around 14 000 μm^3^ and a nuclear volume of around 2 000 μm^3^. The respective volumes were utilized to convert number of molecules per cell into cell type-specific initial concentrations, which are summarized in S1 Table.

To test whether EPO influences survival and may reduce cisplatin-induced apoptosis in NSCLC cells, H838 and H838-HA-hEPOR cells were treated with 5 mg/l of the chemotherapeutic agent cisplatin in combination with 10 U/ml EPO beta or were left untreated for three days. Cell viability was measured every 24 hours by the CellTiter-Blue assay (Fig 1D, S4 Fig). In untreated proliferating H838 and H838-HA-hEPOR cells, a two- to three-fold increase in cell numbers was observed. No significant impact of EPO beta on cell viability was detected during this observation period. The treatment of H838 and H838-HA-hEPOR with 5 mg/l cisplatin reduced cell viability or at least decreased proliferation compared to the untreated control. Interestingly, co-treatment with 10 U/ml EPO beta decreased the impact of cisplatin in H838 and H838-HA-hEPOR at each time point. While there was some variability in the response of these cells to cisplatin, the rescue effect induced by EPO beta was consistent. To validate these findings, the Casper3-GR FRET (fluorescence resonance energy transfer)-based sensor was expressed in H838-HA-hEPOR (H838-HA-hEPOR-Casper3-GR) and caspase-3 activity, a key indicator for apoptotic responses, was measured by life-cell imaging in a time-resolved manner (Fig 1E, S5 Fig). As a positive control 10 μM staurosporine was applied that is known to rapidly induce caspase-3. Indeed, the effect of staurosporine was observed within 5 hours upon treatment whereas in untreated cells no peak of caspase-3 activation was detected. Upon treatment with 5 mg/l cisplatin the maximum peak of caspase-3 activity was reached at around 24 hours followed by a strong signal decrease due to cells undergoing apoptosis. Upon co-treatment with 10 U/ml or 500 U/ml EPO the amplitude of the cisplatin-induced maximal caspase-3 activation showed an EPO dose-dependent reduction, which is in line with the cell viability assay, and a peak shift to around 15 hours. Treatment with EPO alone did not induce caspase-3 activation.

Concluding, we demonstrated that, similar to CFU-E, the EPOR expressed in H838 cells was capable of activating the JAK2/STAT5 signaling cascade. Further, in line with the observation that EPO-induced activation of JAK2/STAT5 signaling correlates with survival signaling [19], we showed in co-treatment experiments that EPO reduces the extent of cisplatin-induced apoptosis in H838 and H838-HA-hEPOR cells and thus exhibits a rescuing effect in the lung cancer cell line and its derivative. Due to the similarity of the core signaling components, it is difficult to predict cell type-specific differences in the signaling network based only on experimental observations. We developed a systematic mathematical modeling approach for the unbiased identification of cell type-specific differences with the aim to propose strategies to exclusively target the lung cancer cells but not the erythroid progenitor cells.

### Development of a strategy to identify cell type-specific parameters

**Fig 2:**
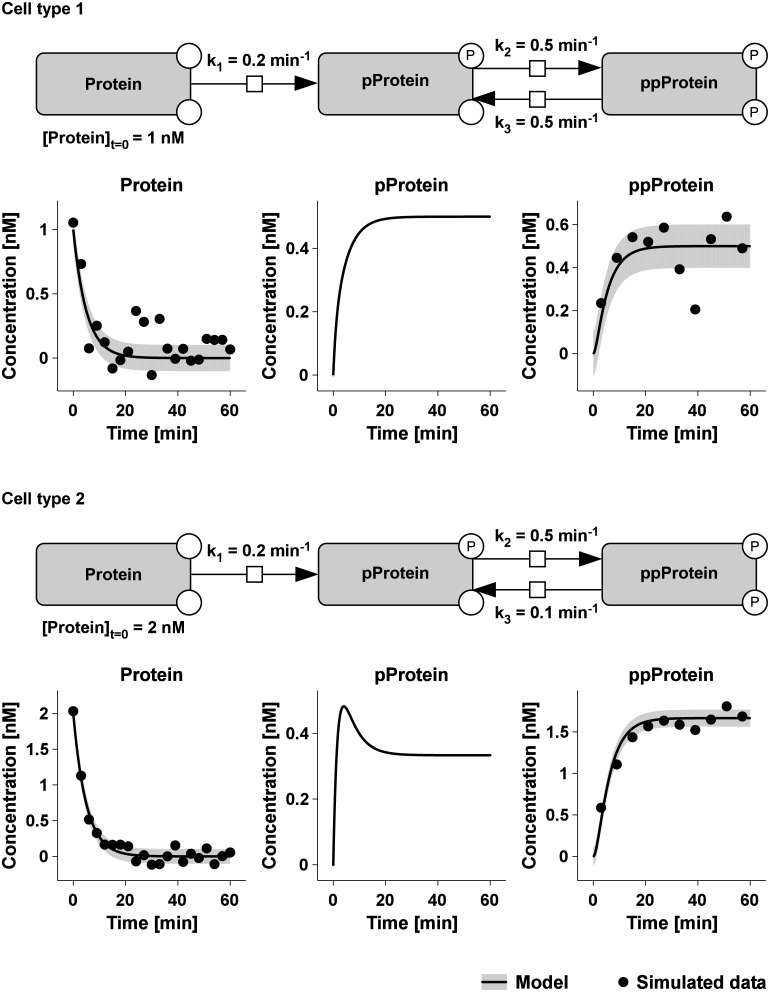
Example model with simulated data for two different cell types. The process diagram of a two-step phosphorylation reaction (Protein→pProtein→ppProtein) is shown according to Systems Biology Graphical Notation. The ODE was numerically solved over time for two cell types that differ in the initial protein concentration ([Protein]_t = 0_) and in one kinetic rate (*k*_3_). The initial concentrations of the phosphorylated compounds were set to zero ([pProtein]_t = 0_ = [ppProtein]_t = 0_ = 0). The second phosphorylation step is reversible and the dephosphorylation rate (k_3_) was assumed to be cell type-specific. The parameters for the phosphorylation steps (*k*_1_, *k*_2_) are the same for both cell types. Simulated data points are depicted as black dots, and the grey shading indicates the standard deviation (σ = 0.1) of the simulated measurement errors. Model trajectories are displayed as black lines.

To predict cell type-specific model parameters between two different cell types, we propose an ODE-based mathematical modeling strategy in combination with a L_1_ regularization method. To show that this approach is capable to identify these parameter differences, first an *in silico* model with simulated data was investigated. The exemplary mathematical model mimics a two-step phosphorylation cascade in two different cell types, in which a protein (Protein) is irreversibly converted into a phosphorylated protein (pProtein) with rate *k*_1_ followed by another phosphorylation step resulting in a doubly phosphorylated protein (ppProtein). This second step can be reversed by a dephosphorylation reaction (Fig 2). The ppProtein and its intermediate pProtein equilibrate depending on the ratio of the forward (*k*_2_) and backward reaction (*k*_3_). The corresponding differential equation system is given by:

$$\begin{array}{cc} \frac{\text{d}\left[Protein\right]}{\text{d}t}= & -k_{1}\cdot \;\left[Protein\right]\\ \frac{\text{d}\left[pProtein\right]}{\text{d}t}= & k_{1}\cdot \left[Protein\right]-k_{2}\cdot \left[pProtein\right]\;+\;k_{3}\cdot \left[ppProtein\right]\\ \frac{\text{d}\left[ppProtein\right]}{\text{d}t}= & k_{2}\cdot \left[pProtein\right]\;-\;k_{3}\cdot \left[ppProtein\right] \end{array}$$

The model structure and the parameters *k*_1_ and *k*_2_ were chosen to be identical for cell type 1 and cell type 2. In contrast, the following two parameters were set as cell type-specific: The rate *k*_3_ was set to 0.5 min^-1^ in cell type 1 and 0.1 min^-1^ in cell type 2 and the parameter [Protein]_t = 0_ was defined as 1 nM in cell type 1 and 2 nM in cell type 2. The initial concentration of pProtein and ppProtein at t = 0 min were assumed to be zero in both cell types.

The ODE solution was calculated using the D2D software [31] and data (black dots) were simulated by adding normally distributed noise (σ = 0.1) (Fig 2). The model trajectories are depicted as black lines and the shading represents the noise distribution. To approximate a realistic setting, only two proteins were assumed to be measurable and sampling time points were not equal. Hence, data was available for Protein (n = 21) and ppProtein (n = 10).

To estimate parameters and identify cell type-specific differences given only by the model structure and the simulated data, the model was parameterized with parameter *p*_i_ for cell type 1 and *r*_*i*_ ∙ *p*_*i*_ for cell type 2, with *r*_*i*_ denoting fold-changes between both cell types. After calculating the maximum likelihood estimates individually for cell type 1 and 2, the regularization weight *λ* of the constrained likelihood

$$C\;=\;L_{\text{cell type 1}}+L_{\text{cell type 2}}+\lambda \sum_{{}_{i}}\left|{\text{log}}_{10}r_{i}\right|$$

was scanned. Here, ℒ_cell type 1_ and ℒ_cell type 2_ denote the likelihood (i.e. the negative two-fold log-likelihood) of the respective cell type and the last term regularizes the fold-changes *r*_*i*_ of parameters between the cell types. If the parameter *p*_*i*_ is the same in both cell types, *r*_*i*_ = 1 and the regularization term is zero. To obtain the regularization path for all parameters, we gradually increased *λ* from 10^−4^ to 10^4^ and re-estimated the constrained likelihood at each step.

**Fig 3:**
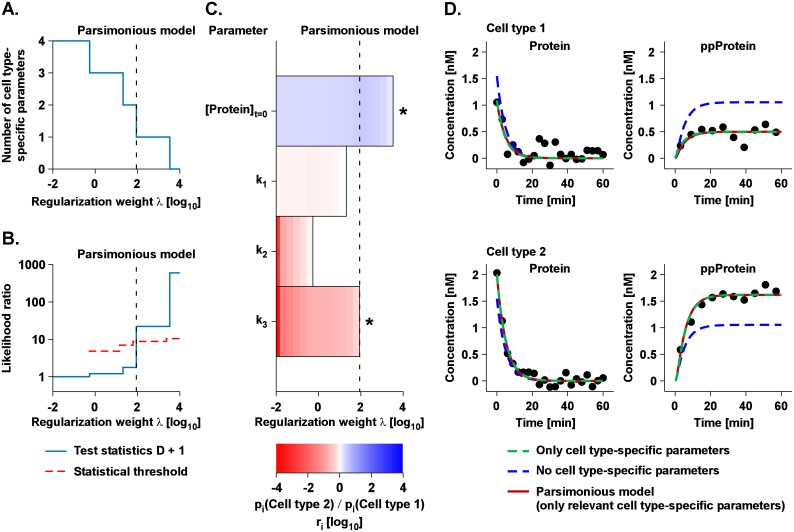
L_1_ regularization recovers cell type-specific parameters. (A) The dependency of the predicted number of cell type-specific parameters and the regularization weight *λ* is shown. (B) The likelihood ratio test was performed for each number of cell type-specific parameters. If the test statistics (blue), i.e. the mismatch between data and model, is larger than the statistical threshold (red dashed), the regularization weight *λ* was too large hence the model was rejected. The crossing of the blue and the dashed red line corresponds to the parsimonious model (dashed black line). (C) The regularization path of the four parameters fold-changes is shown. The regularization-dependent parameter differences are indicated with shades of red (higher in cell type 1) to blue (higher in cell type 2). The asterisks indicate the identified parameter differences. (D) Model trajectories and simulated data for three exemplary scenarios. The dashed green line shows the dynamics for only cell type-specific parameters. In contrast, the blue line displays the model trajectories for no cell type-specific parameters, which is unable to describe the simulated data. Finally, the parsimonious model (solid red) is able to describe the simulated data with only two relevant cell type-specific parameters recovering the simulated parameter differences.

With increasing regularization weight *λ*, the number of cell type-specific parameters decreased until all parameters were independent from the cell type (Fig 3A). To determine the optimal regularization weight *λ*, i.e. to statistically evaluate the minimum number of cell type-specific parameters that are necessary for the model to sufficiently describe the data (parsimonious model), the likelihood ratio test was utilized. The L_1_ regularization was used to select cell type-specific parameters. To reduce bias, the parameters of the non-regularized parsimonious model were then estimated in a second step. The likelihood ratio test statistic *D* = ℒ_*λ*_ − ℒ_full_ was used, where ℒ_full_ denotes the likelihood of the full model with only cell type-specific parameters (*M*) and ℒ_*λ*_ the likelihood of a model with *N* cell type-specific parameters that were selected for a given *λ*. If D < χ^2^_*dof*,*α*_, the smaller model cannot be rejected based on the χ^2^-distribution for a given confidence level *α* and degrees of freedom (*dof* = *M* − *N*).

In Fig 3B, the test statistic *D* is shown by a solid blue line and the statistical threshold based on the likelihood ratio test is shown as a dashed red line. The crossing point of both lines defines the parsimonious model, in which only relevant parameters are defined as cell type-specific. The so-called regularization path shown in Fig 3C indicates the fold-changes between cell type 1 and cell type 2 for all parameters at a given regularization weight. The colors red or blue show whether a parameter is larger in cell type 1 or 2, respectively. The regularization path is also shown in S6A Fig as a line plot. In our example, the parsimonious model that is indicated by the vertical dashed line (Fig 3A, 3B and 3C) has two cell type-specific parameters. These two parameters were the rate *k*_3_ and the initial concentration of Protein [Protein]_t = 0_, which could be reconstructed from the example model as cell type-specific (indicated with asterisks in Fig 3C and S6A Fig). We determined the relative difference and by a profile likelihood approach [35] the corresponding confidence interval (S6B Fig): [Protein]_t = 0_ is larger in cell type 2 by a factor of 1.88 ± 0.17 (true value is 2) and *k*_3_ is larger in cell type 1 by a factor of 4.71 ± 1.20 (true value is 5).

To illustrate how well individual models that differ in the number of cell type-specific parameters can describe the simulated data, model trajectories of three different scenarios were plotted with the simulated data (Fig 3D). The model with only cell type-specific parameters (green dashed line), which is the largest model and has the highest degree of freedom, was capable to describe all data sets. The smallest model with no cell type-specific parameters (blue dashed line) was not in statistical agreement with the simulated data, because e.g. for both cell types the species “ppProtein” was not correctly described. In line with the likelihood ratio test, the parsimonious model with only relevant cell type-specific parameters (solid red line) described the simulated data to a comparable extent as the model with only cell type-specific parameters.

In sum, the L_1_ regularization was able to reconstruct cell type-specific parameters for simulated data using a single scan of the regularization weight *λ*, suggesting that our implementation for dynamic ODE-based mathematical modeling in combination with L_1_ regularization is appropriate to reveal differences between two different cell types.

### Generalized mathematical model structure of the EPO-induced JAK2/STAT5 signaling pathway in CFU-E and H838 cell types

**Fig 4:**
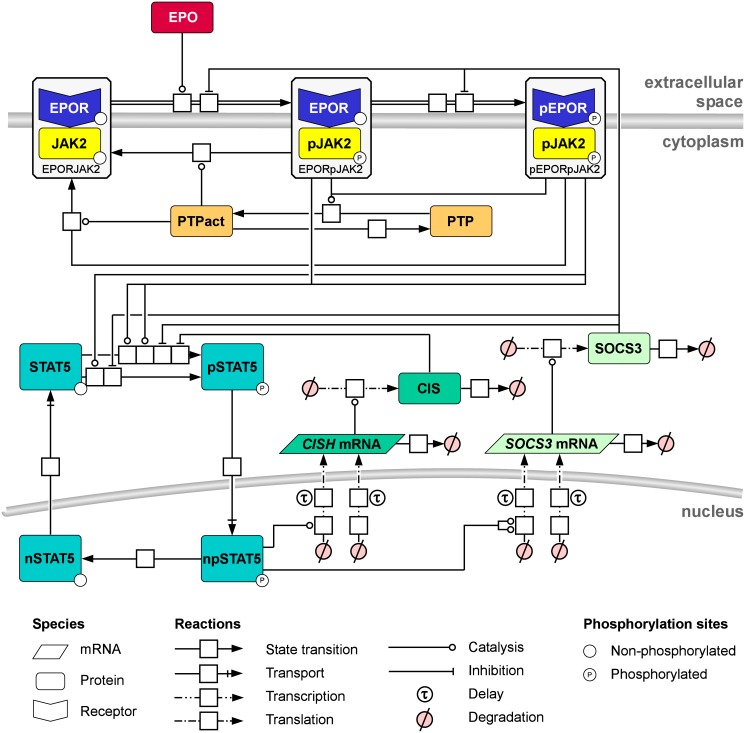
Generalized mathematical model structure of the EPO-induced JAK2/STAT5 signaling pathway. The process diagram of the EPO-induced JAK2/STAT5 signaling pathway model is shown according to Systems Biology Graphical Notation. The binding of the ligand EPO to its cognate receptor results in the phosphorylation of first JAK2 and then of the EPOR (pEPORpJAK2). STAT5 is recruited by pEPOR and phosphorylated by pJAK2 and translocates to the nucleus where it induces the transcription of the negative feedback regulators *CISH* mRNA and *SOCS3* mRNA. Protein tyrosine phosphatase (PTP) regulates the dephosphorylation of the EPOR-JAK2 complex.

To pinpoint cell type-specific differences in EPO-induced JAK2/STAT5 signaling in CFU-E cells and H838 & H838-HA-hEPOR cells by the approach introduced above, the previously published mathematical model of EPO-induced JAK2/STAT5 signaling in murine CFU-E cells [19] was used as reference model structure: The JAK2/STAT5 model for mCFU-E cells described the EPO-induced JAK2 activation in the EPOR-JAK2 complex, the subsequent phosphorylation of the EPO receptor, attenuation by the phosphatase SHP1, the activation of the transcription factor STAT5 by the EPOR-JAK2 complex and the STAT5 transport from cytosol into and out of the nucleus, as well as the induced transcription and translation of the negative feedback regulators SOCS3 and CIS. To describe EPO-induced JAK2/STAT5 signaling in human H838 and H838-HA-hEPOR cells, a generalized model structure was developed by adding the following components to the reference model: (i) Basal RNA activation rates were included, because we observed that basal *CISH* mRNA and *SOCS3* mRNA is also reduced by inhibiting transcription (S7A and S7B Fig). (ii) A dephosphorylation step of nuclear phosphorylated (np) STAT5 was included to be able to distinguish dephosphorylation and nuclear export of STAT5 [36]. (iii) The regulation of the *SOCS3* promotor binding activity by npSTAT5 was described by a Hill coefficient to allow non-linear transcriptional activity. In addition, the receptor phosphatase SHP1 that is known to be restricted to hematopoietic cells was substituted by the general term PTP (Fig 4).

The generalized model structure was parameterized based on cell type-specific quantitative data. First, the model was calibrated by directly implementing cell type-specific quantities for H838 and H838-HA-hEPOR cells such as cellular and nuclear volumes and the initial concentrations of the EPOR as well as JAK2 and STAT5 together with their respective experimental error (summarized in S1 Table). As the ratio of the EPOR and JAK2 was different for H838 and H838-HA-hEPOR cells possibly resulting in a saturation of the phosphatase in H838-HA-hEPOR cells, distinct properties were assumed for the phosphatase PTP in H838-HA-hEPOR cells. Except for this difference and the number of receptors on the surface as shown in S1 Fig, the JAK2/STAT5 signaling models for H838 and H838-HA-hEPOR cells were parameterized identically. Therefore, H838 and H838-HA-hEPOR cells were treated as the same cell type but with different conditions.

**Fig 5:**
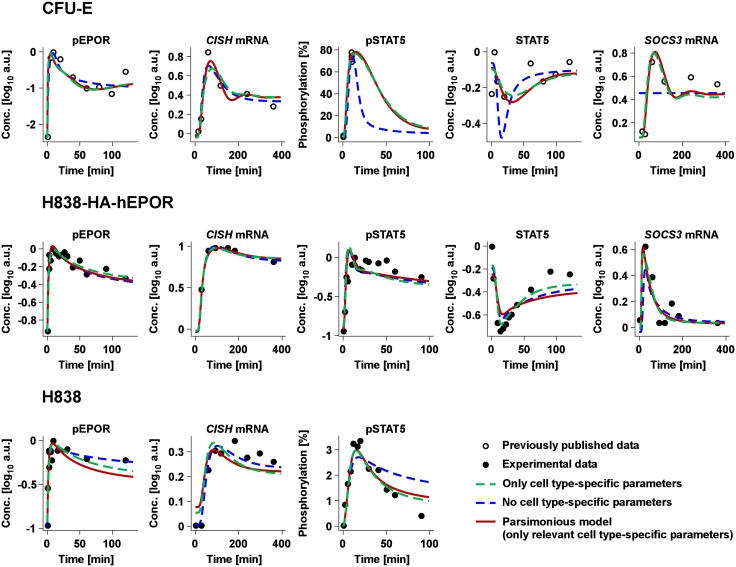
Model selection. The model trajectories for a selection of key pathway components are shown for CFU-E, H838 and H838-HA-hEPOR cells. This includes expression of the EPOR targets *CISH* mRNA and *SOCS3* mRNA measured by qRT-PCR as well as pEPOR, pJAK2 and cytoplasmic STAT5 data measured by quantitative immunoblotting. The amount of pSTAT5 was determined by either mass spectrometry or quantitative immunoblotting. The closed circles represent experimentally measured data in H838 and H838-HA-hEPOR cells. CFU-E data previously published [19] are shown as circles. The lines depict the three applied model strategies: dashed green (only cell type-specific parameters), dashed blue (no cell type-specific parameters) and solid red (parsimonious model, only relevant cell type-specific parameters). The parsimonious model describes the data similarly to the model with only cell type-specific parameters, whereas the trajectories of the model without cell type-specific parameters are not in line with the experimental data, e.g. for *SOCS3* mRNA in CFU-E and for pSTAT5 in H838. All data sets, replicates and trajectories of the parsimonious model are shown in S8 and S9 Figs.

To calibrate the model for CFU-E cells, quantitative data of the reference model [19] was reutilized (open circles in Fig 5). For the NSCLC cell line H838 and its derivative H838-HA-hEPOR, quantitative time-resolved data was generated (black closed circles in Fig 5). For H838 cells, the dynamics of pEPOR, pJAK2 and pSTAT5 and the expression of *CISH* mRNA upon stimulation with 10 U/ml EPO beta were determined. For H838-HA-hEPOR cells, the amount of total and phosphorylated STAT5, pEpoR, pJAK2 and the expression of *CISH* and *SOCS3* mRNA were measured upon stimulation with 10 U/ml EPO beta. While the temporal changes of pEPOR were similar in CFU-E, H838-HA-hEPOR and H838 cells, the dynamics of *CISH* mRNA was more transient in CFU-E cells, while pSTAT5 was more sustained in H838-HA-hEPOR cells than in H838 cells. *SOCS3* mRNA, on the other hand, was more sustained in CFU-E cells than in H838-HA-hEPOR cells. Thus, we observed substantial differences in the dynamics of signaling components in the three cell types analyzed. We performed a multitude of additional measurements with different conditions (S7 Fig). The basal expression levels of *CISH* mRNA and *SOCS3* mRNA were determined for CFU-E and H838-HA-hEPOR cells by applying the transcription inhibitor actinomycin D. Time- and dose-resolved protein quantification was performed by quantitative immunoblotting and relative mRNA quantification by qRT-PCR. The degree of STAT5 phosphorylation (pSTAT5) was measured for both the lung cancer cell line and its derivative by quantitative mass spectrometry.

The generalized model structure comprised 25 kinetic parameters and 3 initial concentrations (the EPOR-JAK2 complex, STAT5 and PTP at time point 0). Assuming that all kinetic parameters and initial concentrations were cell type-specific (i.e. different parameters for CFU-E and for H838 & H838-HA-hEPOR cells), this model was calibrated based on the experimental data of CFU-E (516 data points) using 83 additional observation parameters and of H838 and H838-HA-hEPOR cells (625 data points) using 77 additional observation parameters. For global optimization of the likelihood, a multi-start deterministic strategy was applied [37]. The model could describe all data sets of the cell types tested, shown representatively by the model trajectory of the model with only cell type-specific parameters (dashed green line in Fig 5). Concluding, we identified a model structure that was able to describe the data sets of EPO-induced JAK2/STAT5 signaling in CFU-E, H838 and H838-HA-hEPOR cells.

### L_1_ regularization identifies cell type-specific parameters

**Fig 6:**
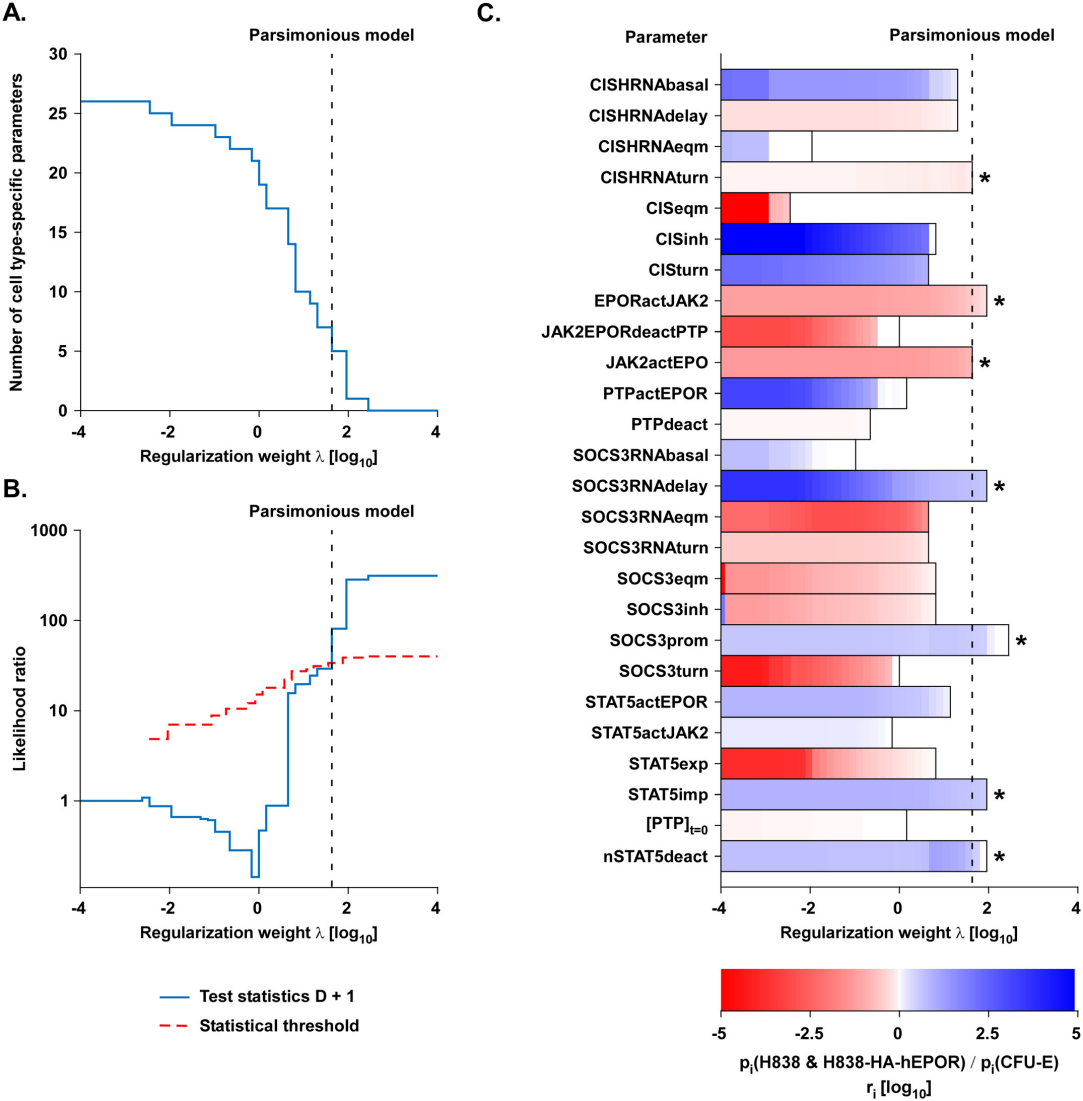
Identification of cell type-specific differences. (A) The number of cell type-specific parameters in dependency of the regularization weight *λ* is shown. (B) The likelihood ratio test statistics was calculated for each regularization weight *λ*, resulting in a nested sub-model with a number of cell type-specific parameters that is smaller compared to the full model. If the test statistics (blue) is larger than the statistical threshold (red dashed), the model reduction step was rejected. The crossing of the blue and the dashed red line corresponds to the parsimonious model (dashed black line). (C) The regularization path of the 26 parameters is shown. The regularization-dependent parameter differences are indicated with shades of red (higher in CFU-E) to blue (higher in H838 & H838-HA-hEPOR). The asterisks depict the identified parameter differences.

Our experimental observations suggested that core reactions of the pathway operate comparable in the different cell systems. We therefore tested whether it is possible to identify cell type-independent and cell type-dependent parameters to establish a parsimonious model. The parsimonious model is defined as the model with the smallest number of cell type-specific parameters that is still compatible with the experimental data. To infer cell type-specific parameters, the L_1_ regularization strategy was applied while parameterizing the mathematical model based on the experimental data established for the different cell types. We tested the 25 kinetic parameters and the initial concentrations of PTP, while the initial concentrations of the EPOR-JAK2 complex and STAT5 were fixed to the measured values (S1 Table). As expected, by increasing the regularization weight the number of cell type-specific parameters was gradually decreased (Fig 6A). In analogy to the example (Fig 3), the parsimonious model was determined using the likelihood ratio test (blue line in Fig 6B). The statistical threshold corresponding to a significance level α = 0.05 is shown by the red dashed line. At the crossing point of these two lines, the parsimonious model with relevant cell type-specific parameters is defined and depicted by the dashed black vertical line in all panels of Fig 6. The parsimonious model with the minimal number of cell type-specific parameters (red line, Fig 5) resulted in trajectories describing the data and showed similar performance as the model with only cell type-specific parameters (dashed green line, Fig 5). On the contrary, the model with only cell type-independent parameters (dashed blue line, Fig 5) was not capable of representing the dynamics of the data as evidenced e.g. by the difference of the model trajectory and the experimental data of *SOCS3* mRNA. The parsimonious model simultaneously described all experimental data measured in CFU-E (S8 Fig), H838-HA-hEPOR (S9A and S9B Fig) and H838 (S9B and S9C Fig).

Seven relevant cell type-specific parameters are highlighted with an asterisk in the regularization path of the 26 parameters in Fig 6C and in an alternative representation in S10A Fig. We ensured significance by calculating the confidence intervals of these parameter differences (S10B Fig). Table 1 provides a description of the 26 parameters and the model-predicted differences between CFU-E cells and H838 & H838-HA-hEPOR cells. The cell type-specific parameters predicted to have higher values in CFU-E cells (depicted in red, Fig 6C) were the *CISH* mRNA turnover rate (CISHRNAturn), the activation rate of the EPOR by JAK2 (EPORactJAK2) and the activation rate of JAK2 by EPO (JAK2actEPO). In contrast, the parameters that were predicted to have higher values in the H838 & H838-HA-hEPOR cells (depicted in blue, Fig 6C) were the parameter inversely defining the delay in *SOCS3* mRNA production (SOCS3RNAdelay), the *SOCS3* promoter activity (SOCS3prom) the import rate of pSTAT5 into the nucleus (STAT5imp) and the deactivation rate of STAT5 in the nucleus (nSTAT5deact).

**Table 1:** Parameters and identified cell type-specific differences.

Name	Description	> in H838 & H838-HA-hEPOR	> in CFU-E	Factor
CISHRNAbasal	Basal production rate of *CISH* mRNA			
CISHRNAdelay	Delay parameter for *CISH* mRNA production			
CISHRNAeqm	*CISH* mRNA equilibrium concentration			
CISHRNAturn	*CISH* mRNA turnover rate		*	2.4 ± 0.5
CISeqm	CIS equilibrium concentration			
CISinh	Inhibition strength imposed by CIS			
CISturn	CIS turnover rate			
EPORactJAK2	Activation rate of EPOR by pJAK2		*	12 ± 4
JAK2EPORdeactPTP	Deactivation rate of JAK2 and EPOR by PTP			
JAK2actEPO	Activation rate of JAK2 by EPO		*	16 ± 3
PTPactEPOR	Activation rate of PTP by EPOR			
PTPdeact	Deactivation rate of PTP			
SOCS3RNAbasal	Basal production rate of *SOCS3* mRNA			
SOCS3RNAdelay	Delay parameter for *SOCS3* mRNA production	*		≥ 6.5
SOCS3RNAeqm	*SOCS3* mRNA equilibrium concentration			
SOCS3RNAturn	*SOCS3* mRNA turnover rate			
SOCS3eqm	SOCS3 equilibrium concentration			
SOCS3inh	Inhibition strength imposed by SOCS3			
SOCS3prom	*SOCS3* promoter activity	*		3.7 ± 0.5
SOCS3turn	SOCS3 protein turnover rate			
STAT5actEPOR	Activation rate of STAT5 by pEPOR			
STAT5actJAK2	Activation rate of STAT5 by pJAK2			
STAT5exp	Export rate of STAT5 from nucleus			
STAT5imp	Import rate of STAT5 to nucleus	*		4.9 ± 0.7
[PTP]_t = 0_	Initial PTP concentration			
nSTAT5deact	Deactivation rate of npSTAT5 in nucleus	*		12 ± 4

The parameter that showed strongest evidence for a difference between both cell types was the parameter regulating npSTAT5-induced *SOCS3* promoter activity (SOCS3prom) (Fig 6C). The model predicted a linear correlation of the *SOCS3* mRNA production rate and the concentration of npSTAT5 for CFU-E cells (S11A Fig). In contrast, the same prediction analysis for H838 & H838-HA-hEPOR cells indicated a threshold behavior of the *SOCS3* mRNA production rate with respect to npSTAT5. The analysis showed that in these cells a certain amount of activated STAT5 has to be present in the nucleus to induce *SOCS3* mRNA synthesis (S11B Fig). To test whether this difference between cell types can be explained by differences in promoter binding elements or epigenetic modifications, the human and murine *SOCS3* promoter was analyzed for STAT5 binding sites. Indeed, the murine promoter contains four STAT5 binding sites, whereas the human harbors only two (S11C Fig). Additionally, we performed methylation analyses and demonstrated that the *SOCS3* promoter is accessible in both CFU-E and H838 cells (S11C Fig).

Taken together, the selected parsimonious model could simultaneously describe the data sets of CFU-E, H838 and H838-HA-hEPOR cells. Seven relevant cell type-specific parameters were identified and three of these parameters had higher values in CFU-E cells and four had higher values in H838 & H838-HA-hEPOR cells.

### Validation of the predicted cell type-specific models

The parsimonious model predicted several cell type-specific parameters comprising mRNA processing: the turnover rate of the *CISH* mRNA, the *SOCS3* promoter activity and the *SOCS* mRNA delay parameter. Therefore, we selected mRNA processing as a biological process that can be measured experimentally and is connected to parameters identified by the model as cell type-specific for experimental validation of the parsimonious model. We hypothesized that by the application of the transcriptional inhibitor actinomycin D the dynamics of *CISH* mRNA and *SOCS3* mRNA would be altered in a cell type-specific manner.

**Fig 7:**
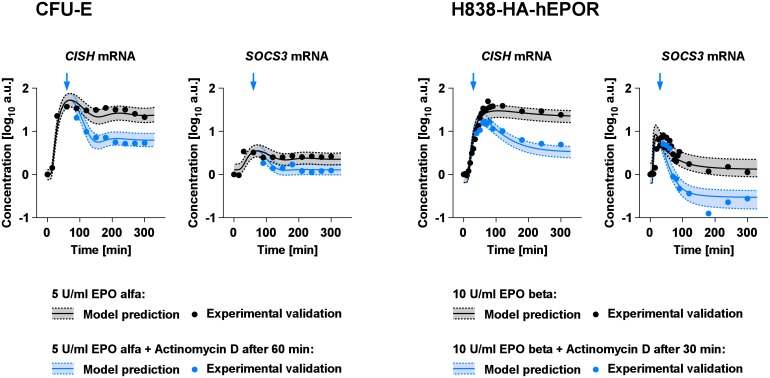
Experimental validation of the cell type-specific *CISH* and *SOCS3* mRNA parameters. The parsimonious model was employed to predict the dynamics of *CISH* mRNA and *SOCS3* mRNA upon treatment with a transcriptional inhibitor. The mRNA dynamics in CFU-E stimulated with 5 U/ml EPO alfa alone (black) or with transcriptional inhibition after 60 min (blue) was predicted. Additionally, the mRNA dynamics in H838-HA-hEPOR stimulated with 10 U/ml EPO beta alone (black) or with transcriptional inhibition after 30 min (blue) was predicted. Shadings surrounded by dotted lines depict uncertainty of the prediction. CFU-E cells were stimulated with 5 U/ml EPO alfa and either additionally treated with 1 μg/ml actinomycin D, to inhibit transcription, at 60 min (blue arrows) or left untreated. The H838-HA-hEPOR cells were either stimulated with 10 U/ml EPO beta alone (black) or additionally with 1 μg/ml actinomycin D at 30 min (blue arrows). The mRNA was extracted at the indicated time and the *SOCS3* and *CISH* mRNA levels were measured with qRT-PCR. Experimental data are depicted as closed circles. The experiment was performed in triplicates and one representative example is shown.

First, the dynamics of *CISH* mRNA and *SOCS3* mRNA were predicted for CFU-E and H838-HA-hEPOR cells for 300 min upon treatment with EPO (black) or EPO in combination with actinomycin D (blue) (Fig 7). We proposed by experimental design that the transcriptional inhibitor should be applied at the predicted peak of mRNA expression upon EPO stimulation, which was after 60 min for CFU-E cells treated with 5 U/ml EPO alfa and after 30 min for H838-HA-hEPOR stimulated with 10 U/ml EPO beta (blue arrows in Fig 7). To assess the uncertainty of the model dynamics, the prediction profile likelihood [38, 39] was utilized. The shading with dotted lines indicates the 1σ confidence interval. The EPO-induced dynamic of *CISH* mRNA and *SOCS3* mRNA was predicted to show a transient peak followed by a new steady state in both cell types. Addition of actinomycin D was predicted to result in a more dramatic decrease after the treatment and a steady state that is lower compared to EPO treatment alone. The *SOCS3* mRNA in H838-HA-hEPOR cells was predicted to even decrease below the starting level after around two hours.

To experimentally validate these predictions, H838-HA-hEPOR and CFU-E cells were treated either with 10 U/ml EPO beta or 5 U/ml EPO alfa alone (black dots) or in combination with 1 μg/ml actinomycin D (blue dots) at the predicted time points of either 60 min (CFU-E) or 30 min (H838-HA-hEPOR). The dynamics of *CISH* mRNA and *SOCS3* mRNA were measured by qRT-PCR. Unknown experiment-specific offset and scaling parameters were estimated from this validation data. As shown in Fig 7, the experimentally measured *CISH* mRNA and *SOCS3* mRNA (dots) were in line with the model predictions (lines with shadings).

In summary, the model predicted cell type-specific differences in the dynamics of the *SOCS3* and *CISH* mRNA could be experimentally validated. Therefore, the parsimonious model and the uncovered cell type-specific differences were used to recommend strategies to specifically target JAK2/STAT5 signaling in H838 cells.

### Mathematical model predicts cell type-specific drug targets

To identify potential targets in the EPO-induced JAK2/STAT5 signaling pathway, a sensitivity analysis was performed for CFU-E and H838 cells. The area-under-curve of npSTAT5 at 60 min was taken as read out (*Κ*), because we previously could correlate this to survival of CFU-E cells [19]. A control coefficient

$$S_{p_{i}}^{K}\;=\;\frac{p_{i}}{K}\cdot \frac{\partial K}{\partial p_{i}}$$

was calculated for each model parameter (*p*_*i*_) in both cell types [23]. The larger the absolute value of the control coefficient, the larger is the influence of a parameter to the simulated biological readout. If a control coefficient is positive, a decrease of the parameter induces a decrease in the area-under-curve of npSTAT5. Interestingly, we observed not only that several parameters exerted major control over npSTAT5, but also that the control coefficients of these parameters differed between CFU-E and H838 cells (S12 Fig). We identified six parameters that had a larger control coefficient for JAK2/STAT5 signaling in H838 cells than on CFU-E cells and thus could represent suitable therapeutic targets (indicated with asterisks in S12 Fig): The activation rate of STAT5 by pEPOR, the activation rate of JAK2 by EPO, the activation rate of STAT5 by pJAK2, the activation rate of the EPOR by pJAK2, the *SOCS3* mRNA turnover rate and the deactivation rate of PTP. The inhibition of these parameters would diminish the npSTAT5 level and thus the STAT5-mediated survival in the H838 cells more than in CFU-E cells.

**Fig 8:**
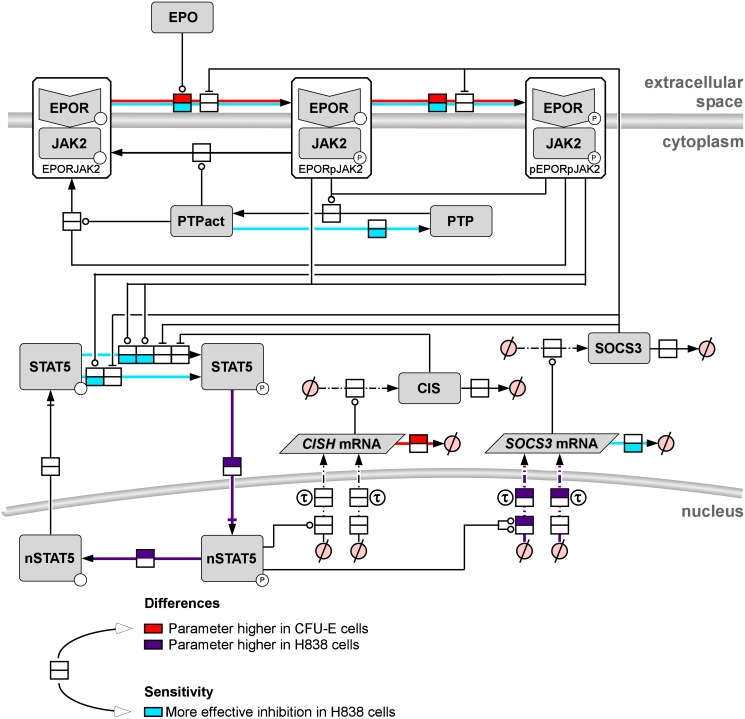
Sensitivity and differences. The process diagram of the EPO-induced JAK2/STAT5 signaling pathway model is shown according to Systems Biology Graphical Notation. Identified parameter fold-changes between CFU-E and H838 cells are shown in red (higher in CFU-E: JAK2actEPO, EPORactJAK2, CISHRNAturn) or purple (higher in H838: STAT5imp, nSTAT5deact, SOCS3prom, SOCS3RNAdelay). Parameters with a more effective inhibition in H838 cells are shown in light blue (JAK2actEPO, EPORactJAK2, STAT5actJAK2, STAT5actEPOR, STAT5imp, STAT5exp, SOCS3RNAturn). The area-under-curve of npSTAT5 at 60 min after stimulation was used as read-out to calculate the sensitivities.

The cell type-specific parameters and the predicted therapeutic targets are summarized by a color code in the process diagram of the JAK2/STAT5 pathway (Fig 8). Cell type-specific differences in the parameter values are depicted in the upper part of each reaction square: Red indicates that the parameter had higher values in CFU-E cells, purple that the parameter value was higher in H838 and H838-HA-hEPOR cells and colorless that there is no difference between cell types. The predicted therapeutic targets are marked in blue in the lower part of each reaction square. Most of the differences were related to the receptor-kinase complex and the shuttling of STAT5. Interestingly, the potential drug targets identified by the sensitivity analysis did not entirely overlap with cell type-specific differences in parameter values. Parameters with a higher control coefficient in H838 cells than in CFU-E cells were either related to the activation of the JAK2-EPOR complex and its control of STAT5 phosphorylation or *SOCS3* mRNA degradation. The shuttling of STAT5 was in fact cell type-specific, but the control coefficient had a higher value in CFU-E cells (S12 Fig), meaning that CFU-E cells would be particularly sensitive for inhibitors and npSTAT5 levels would decrease in the erythroid progenitor cells and not, as intended, in the lung cancer cells.

Based on these results, the model suggests inhibitors for the receptor complex, e.g. a JAK2 inhibitor, as optimal drugs to diminish the EPO-induced JAK2/STAT5 signaling specifically in H838 cells while the CFU-E cells would continue to benefit from EPO treatment.

In sum, we demonstrate that the approach of ODE-based mathematical modeling in combination with L_1_ regularization is an appropriate method to identify cell type-specific differences and suitable therapeutic targets.

## Discussion

In this study, we present an approach that combines ODE-based mathematical modeling with L_1_ regularization to identify differences in the EPO-induced JAK2/STAT5 signaling pathway between the NSCLC cell line H838 and CFU-E cells. Based on these differences, targeted inhibitor treatments are predicted to reduce EPO-induced survival signaling in the lung cancer cells with only marginally affecting EPO-induced signaling in healthy erythroid progenitor cells.

We demonstrated that the EPOR is expressed in the NSCLC cell line H838, is phosphorylated upon EPO stimulation and activates JAK2/STAT5 signaling associated with cell survival. We verified that EPO inhibits cisplatin-induced apoptosis in H838 cells. This indicates that EPO might not only have an effect on erythroid progenitor cells, but also could induce EPO-mediated survival signaling in other cells expressing the EPOR, including tumor cells. This effect has been previously observed in other cancer entities: It was shown that EPOR signaling affected survival of EPOR expressing melanoma cells *in vitro* and in xenograft mice [14] and that EPO activates survival pathways in breast cancer stem-like cells and increases the resistance to chemotherapeutic agents [40]. Having demonstrated that EPO potentially targets not only CFU-E cells but also lung cancer cells, it remained to be addressed if the signaling upon EPO stimulation is identical between the different cell types or if there is a therapeutic window to specifically target lung cancer cells.

We quantitatively analyzed EPO-induced JAK2/STAT5 signaling in CFU-E, H838 and H838-HA-hEPOR cells and identified cell type-specific differences in the activation dynamics of the EPOR and STAT5 as well as the induction of *CISH* mRNA and *SOCS3* mRNA. For CFU-E cells, we previously showed by mathematical modeling of the JAK2/STAT5 signaling pathway that *CISH* mRNA attenuates STAT5 activation primarily at low EPO levels, while *SOCS3* mRNA reduces STAT5 phosphorylation at high EPO concentrations. Importantly, with this approach we quantitatively linked STAT5 phosphorylation to survival of CFU-E cells [41].

Previously, differences between cell types have been identified based on either differential gene expression or genomic mutations. The advantage of the mainly array- or sequencing-based methods is the simultaneous analysis of multiple genomic alterations. For example, a statistical deconvolution approach to derive the relative mRNA abundance for each cell type and infer the relative cell type frequencies from microarray data of mixed tissue samples has been reported [42]. A pioneering sequencing study analyzing samples of 17 NSCLC patients has revealed copy number alterations in the EPOR gene (one patient), mutations in the EPOR gene (two patients) and mutations in the JAK2 gene (two patients) [43]. These snapshot data yield static information on cellular differences, while our time- and dose-resolved measurements additionally provide insights into cell type-specific dynamic properties of the signaling pathways. Furthermore, it is unclear how mutations and copy number alterations translate into dynamic properties such as signaling behavior and cell fate decisions. An important difference affecting the dynamics of signaling pathways in individual cell types is the cellular concentrations of the involved proteins. Recently, by a mass spectrometric approach based on the proteomic ruler, protein abundances in primary human hepatocytes and the human hepatoma cell line HepG2 were compared, revealing that uptake transporters and phase I enzymes were either absent or expressed in very low amounts in HepG2 cells [44]. Also by mass spectrometry, differences in the protein amounts between primary mouse hepatocytes and the murine hepatoma cell line Hepa1-6 were identified [45]. Such results can be readily combined with our L_1_ approach. We have estimated the initial concentrations of the EPOR-JAK2 complex and STAT5 based on our quantitative immunoblotting results, while differences in the initial concentration of PTP were tested by L_1_ regularization. Our results revealed that the concentration of PTP was not decisive for the differences between CFU-E cells and H838 cells.

In this study, we particularly considered the signaling components of the EPO-induced signaling pathway that are essential to describe the dynamics of the JAK2/STAT5 pathway by ODEs and identified parameters that are cell type-specific for either H838 or CFU-E cells by using L_1_-regularized optimization. After the cell type-specific parameters were identified, a non-regularized optimization was performed to calculate unbiased final parameter values. This procedure estimates parameter differences in analogy to classical backward elimination, which constitutes a major difference to lasso where regularization is used for parameter selection as well as to reduce variance by introducing bias (shrinkage) [25]. In such a statistical application, the main goal is typically to minimize prediction error, while the parameter values are not important *per se*. In our case, we were additionally interested in the relative differences of these parameter values. Here, we for example inferred that the import rate of STAT5 is five times larger in CFU-E cells than in H838 cells. Therefore, we used L_1_ regularization only to suggest a set of parameters that were cell type-specific and then calculated the final parameter estimates with a non-regularized optimization.

The seven predicted cell type-specific parameters comprised the *CISH* mRNA turnover rate, the activation rate of the EPOR by JAK2, the activation rate of JAK2 by EPO, the delay in *SOCS3* mRNA production, the *SOCS3* promotor activity, the import rate of pSTAT5 into the nucleus, and the deactivation rate of nuclear pSTAT5. We identified faster activation rates of JAK2 by EPO and of the EPOR by pJAK2 in CFU-E than in H838 and H838-HA-hEPOR cells. This might be due to differences in the affinity of the human-derived EPO to the murine EPOR in CFU-E cells. Additionally, JAK2 is highly expressed in CFU-E cells, while the JAK2 concentration is limiting in H838 cells (S1 Table). It was proposed that JAK2 acts as a chaperone that binds to the EPOR in the endoplasmic reticulum and thereby enhances cell surface expression of the receptor [46]. The import rate of pSTAT5 to the nucleus and the deactivation rate of npSTAT5 were predicted to be higher in H838 and H838-HA-hEPOR cells. It was previously suggested that a cell with a smaller nucleus imports cargo faster [47]. While H838 and H838-HA-hEPOR have a larger nucleus than CFU-E cells, the nucleus to cell volumetric ratio is 0.17 in these cells compared to a ratio of 0.69 in CFU-E cells. The deactivation rate of nuclear pSTAT5 is controlled by nuclear phosphatases. The small dual-specificity phosphatase VHR has been identified to dephosphorylate IFNβ-induced pSTAT5 in the nucleus [48]. It remains to be shown if the same phosphatase dephosphorylates EPO-induced pSTAT5 and whether this phosphatase is differentially expressed in a cell type-specific manner. The model further identified that the parameter for the *SOCS3* promoter activity had a higher value in H838 and H838-HA-hEPOR cells. This parameter resulted in a linear dependency between pSTAT5 concentration and *SOCS3* mRNA expression in CFU-E cells. On the other hand, in H838 and H838-HA-hEPOR cells *SOCS3* mRNA was only expressed if the pSTAT5 concentration in the nucleus exceeded a certain threshold. To analyze the underlying mechanism, we compared the structure of the human and murine *SOCS3* promoters, revealing the presence of additional STAT5 binding sites in the murine promoter, which potentially strengthen the link between transcription factor concentration and mRNA expression. Previously, hypermethylation in CpG islands of the *SOCS3* promoter in H838 correlating with transcriptional silencing was reported [49]. However, our analyses in CFU-E and H838 cells demonstrated low promoter methylation levels in both cell types, indicating that the *SOCS3* promoter should be accessible for the transcriptional machinery in both cell types. The remaining two differential parameters concern mRNA processing: The *CISH* mRNA turnover rate has higher values in CFU-E and the delay parameter for *SOCS3* mRNA production has higher values in H838 and H838-HA-hEPOR cells. Turnover rates of mRNAs can be controlled by the decay rates influenced by both mRNA sequence elements and cellular factors, as reviewed previously [50]. The delay parameter for *SOCS3* mRNA production summarizes transcription, pre-mRNA processing and export to the cytoplasm. In line with our results, it was shown by global run-on sequencing that transcription rates not only vary between different genes, but that they can vary between identical genes in different cell types [51]. Of the seven identified cell type-specific differences, three were associated with mRNA induction and processing. We were able to predict the distinct cell type-specific dynamics of EPO-induced *CISH* mRNA and *SOCS3* mRNA production and mRNA degradation in response to actinomycin D inhibition. Our experimental results confirmed these model predictions.

To predict potential drug targets that primarily affect one cell type, we performed a sensitivity analysis. Since we aimed to diminish the EPO-induced STAT5-mediated survival signal in the lung cancer cells, we focused on potential targets for more effective inhibition in H838 cells. Interestingly, we identified that parameter differences and differential sensitivities do not entirely coincide. On the one hand, the activation rates of JAK2 by EPO and of the EPOR by pJAK2 are faster in CFU-E cells but can be targeted more efficiently in H838 cells. On the other hand, the activation rates of STAT5 by the EPOR-pJAK2 and by the pEPOR-pJAK2 complexes, while associated with the same parameter values in CFU-E and H838 cells, largely affect the pSTAT5 output in H838 cells and have only minor control in CFU-E cells. Other examples for cell type-independent rates but higher control coefficients in H838 cells include the *SOCS3* mRNA turnover rate and the PTP deactivation rate. We previously performed mathematical modeling of the EPO-induced JAK2/STAT5 signaling in BaF3-mEPOR cells, a hematopoietic cell line that is a frequently used model system to study EPOR signaling, and we showed that the parameters of nuclear shuttling are most sensitive to perturbation [36]. Here, we identified STAT5 shuttling parameters to be cell type-specific. Additionally, the control coefficients of the pSTAT5 import and pSTAT5 export parameters have higher values in CFU-E cells than in H838 cells, indicating that the sensitivity of nuclear STAT5 shuttling seems to be restricted to cells with a larger nuclear to cytoplasmic ratio such as CFU-E cells. The observed incongruity of parameter differences and differential sensitivity has previously been predicted by theoretical approaches. It was proposed that kinases that are mutated tend to lose part of their control on signaling, while some of the non-mutated genes may become more important [52]. Concluding, it is not only important to identify which parameters are different, but also a mathematical model is necessary to understand how these differences affect cell type-specific intervention points.

We identified possibilities for cell type-specific targets. Since we compared murine CFU-E cells to human H838 cells, we cannot entirely exclude that some of the predicted differences are species-related. Therefore, validation of the predicted drug targets in human CFU-E cells would be advantageous. Of the six predicted targets for more effective inhibition in H838 cells, the JAK2-mediated reactions are most promising. The model suggested that JAK2 inhibition in combination with EPO treatment affects lung cancer cells to a much higher extent than erythroid progenitor cells. Several JAK2 inhibitors have been developed, including Fedratinib and Ruxolitinib. Preclinically, it was observed by injection of MMTV-Wnt-1 tumor cells into mammary fat pads of mice that inhibition of EPO-induced JAK2 activation by Fedratinib was synergistic with chemotherapy for breast tumor-initiating cells [53]. Clinically, therapy of myelofibrosis with Fedratinib showed beneficial efficacy. However, severe toxic effects in some patients were observed and clinical development of Fedratinib was therefore discontinued [54]. On the other hand, the JAK2 inhibitor Ruxolitinib has been approved for the treatment of myelofibrosis in the United States and in the European Union [55]. Also for solid tumors, the benefit of several JAK inhibitors is currently investigated in clinical studies, including a phase II study with Ruxolitinib in combination with pemetrexed/cisplatin in NSCLC [56].

Concluding, we anticipate that both basic and translational research will benefit from the proposed strategies to identify cell type-specific differences and to predict drug targets that affect cancer cells without impairing healthy cells.

## Materials and Methods

### NSCLC cell line and its derivative and cell culture conditions

Human lung adenocarcinoma cell line H838 was purchased from ATCC (CRL-5844) and cultivated in Dulbecco's modified Eagle's Medium (DMEM, Lonza) supplemented with 10% fetal calf serum (Gibco), 100 μg/ml streptomycin (Gibco) and 100 U/ml penicillin (Gibco). The Phoenix ampho packaging cell line [57] was cultured in DMEM (Gibco) supplemented with 10% fetal calf serum, 100 μg/ml streptomycin (Gibco) and 100 U/ml penicillin (Gibco). For the EPOR overexpressing cell line (H838-HA-hEPOR) 1.5 μg/ml puromycin (Sigma) was added. As growth factor depletion medium DMEM without phenol-red (Lonza) supplemented with 1 mg/ml BSA (Sigma), 100 μg/ml streptomycin (Gibco), 100 U/ml penicillin (Gibco) and 2 mM L-glutamine (Gibco) was used. All cells were cultivated at 37°C, 5% CO_2_ and 95% relative humidity.

### Generation of stably transduced H838 cells

Generation of the retroviral expression vectors pMOWS-GFP [58] and pMOWS-HA-hEPOR [59] have previously been described. To generate pMOWS-Casper3-GR, the Casper3-GR cassette of the vector pCasper3-GR (evrogen, #FP971) was cut out with the restriction enzymes BamHI and NotI and subcloned into the retroviral expression vector pMOWS [58], in which the puromycin resistance cassette was replaced with a neomycin resistance cassette.

Transfection of Phoenix ampho cells was performed by calcium phosphate precipitation. Transducing supernatants were generated 24 hours after transfection by passing through a 0.45 μm filter and supplemented with 8 μg/ml polybrene (Sigma). Stably transduced H838 cells were selected in the presence of 1.5 μg/ml puromycin (Sigma) 48 hours after transduction for H838-GFP and H838-HA-hEPOR and for the H838-HA-hEPOR-Casper3-GR cell line in the presence of additional 400 μg/ml G418 (Sigma).

Surface expression of the EPOR in H838-HA-hEPOR cells was verified by flow cytometry. H838-HA-hEPOR cells were detached with Cell Dissociation Solution (Sigma) according to the manufacturer’s instructions and stained with anti-HA antibody (Roche) diluted 1:40 in 0.3% PBS/BSA for 20 min at 4°C. Cells were washed with 0.3% PBS/BSA and incubated with secondary Cy5-labeled antibody against rat (Jackson Immuno Research), diluted 1:100 in 0.3% PBS/BSA, for 20 min at 4°C in the dark. After washing samples with 0.3% PBS/BSA, propidium iodide (BD Biosciences) was added to exclude dead cells. Canto II (BD Bioscience) was used for sample analysis. The expression of the Casper3-GR sensor and GFP was verified by life cell imaging.

### Isolation of TER119^−^ erythroid progenitor cells at the CFU-E stage from murine fetal livers

All animal experiments were approved by the governmental review committee on animal care of the state Baden-Württemberg, Germany (reference number DKFZ215). At E13.5 Balb/c mouse embryos were dissected from the uteri of female mice euthanized by CO_2_ inhalation. Fetal livers were resuspended in PBS/0.3% BSA and passed through a 40-μm cell strainer (BD Biosciences). Fetal liver cells (FLCs) were treated with 9 ml Red Blood Cell Lysis Buffer (Sigma-Aldrich) to remove erythrocytes. For sorting TER119^−^ erythroid progenitors, FLCs were incubated with rat antibodies against the following surface markers: GR1, CD41, CD11b, CD14, CD45R/B220, CD4, CD8 and Ter119 (BD Pharmingen), and 42.2.2 for 30 min at 4°C. After washing, cells were incubated for 30 min at 4°C with anti-rat antibody-coupled magnetic beads and negatively sorted with MACS columns according to the manufacturer's instructions (Miltenyi Biotech). Sorted CFU-E cells were cultivated for 12–14 hours in Panserin 401 (PAN-biotech) supplemented with 50 μM β-mercaptoethanol and 0.5 U/ml EPO alfa. Before the experiments, cells were growth factor-depleted for 60 min.

### RNA extraction, cDNA synthesis and quantitative Real-Time-PCR

Total RNA was extracted using the miRNeasy Mini Kit (QIAGEN) according to the manufacturer’s instructions. cDNA was generated from 1 μg of total RNA using High Capacity cDNA Reverse Transcription Kit (Applied Biosystems) according to the manufacturer’s instructions. cDNA templates were analyzed by quantitative Real-Time-PCR (qRT-PCR) on a LightCycler 480 (Roche Applied Science) cycler using the LightCycler 480 Probes Master with final 0.4 μM primer and 0.2 μM FAM-labeled hydrolysis probes (Universal Probe Library, Roche Applied Science). Crossing point values were calculated using the second-derivative-maximum method of the LightCycler 480 Basic Software (Roche Applied Science). Quantitative RT-PCR efficiency correction was performed for each setup individually. Concentrations were normalized using the geometric mean of β-glucuronidase (*GUSB*) and esterase D (*ESD*) for the NSCLC cell line and its derivative and hypoxanthine-guanine phosphoribosyltransferase (*HPRT*) for the CFU-E cells. Primers were designed using the UniversalProbe Library Assay Design Center (Roche Applied Science).

UPL Probes and primer sequences for murine samples were CISH_for 5′-gacatggtcctttgcgtaca-3′ CISH_rev 5′-atgccccagtgggtaagg-3′ probe#1, SOCS3_for 5′- gctggtactgagccgacct-3′ SOCS3_rev 5′-aacttgctgtgggtgaccat-3′ probe#83, and HPRT_for 5′-tcctcctcagaccgctttt-3′ HPRT_rev 5′-cctggttcatcatcgctaatc-3′ probe#95. UPL Probes and primer sequences for human samples were CISH_for 5′-agccaagaccttctcctacctt-3′ CISH_rev 5′-tggcatcttctgcaggtgt-3′ probe#20, SOCS3_for 5′-agacttcgattcgggacca-3′ SOCS3_rev 5′-aacttgctgtgggtgacca-3′ probe#36, ESD_for 5′-ttagatggacagttactccctgataa-3′ ESD_rev 5′-ggttgcaatgaagtagtagctatgat-3′ probe#27 and GUSB_for 5′-cgccctgcctatctgtattc-3′ GUSB_rev 5′-tccccacagggagtgtgtag-3′ probe#57.

### CellTiter-Blue viability assay

H838 and H838-HA-hEPOR cells were seeded in a 96-well plate at a density of 10 000 cells/well for three days. Before the experiment, cells were growth factor-depleted for 14–16 hours. To measure the viability of cells, CellTiter-Blue Viability Assay (Promega) was applied according to the manufacturer’s instructions. Incubation with the dye for 60 min was followed by measurement of the fluorescence with the infinite F200 pro Reader (TECAN). A blank well containing culture medium but no cells was measured as background.

### Caspase-3 apoptosis assay

For the caspase-3 activity assay, H838 cells overexpressing HA-hEPOR and additionally expressing the FRET-based Casper3-GR sensor (H838-HA-hEPOR-Casper3-GR) were seeded in 8-well plates at a density of 20 000 cells/well. 24 hours after seeding, cells were growth factor-depleted for 16 hours and then treated with 5 mg/l cisplatin (Teva) or left untreated. Cells were imaged on an environment-controlled microscope (Zeiss LSM 710) and GFP and RFP intensity was determined. Images were acquired every 20 min for 64 hours.

### Determination of cellular and nuclear volumes

H838-GFP cells were amplified to a density of 80% and growth factor-depleted for 14–16 hours. The cells were trypsinized with 0.025% Trypsin/EDTA/PBS (Invitrogen) for 5 min and resuspended in DMEM (Lonza) and Hoechst (H33342) (final concentration 1 μg/ml) was added. The cells were imaged on an environment-controlled microscope (Zeiss LSM 710) and the data was analyzed with Fiji software [60].

### Quantitative immunoblotting

For the detection of the EPOR, JAK2 and STAT5, 800 000 H838 and H838-HA-hEPOR cells, respectively, were seeded three days in advance in a 10-cm plate and washed three times with DMEM without additives and then kept for 3 hours in DMEM with 1% penicillin/streptomycin, 2 mM L-glutamine (Gibco) and 1 mg/ml BSA. The cells were stimulated with 10 U/ml EPO beta (Roche) and lysed with 500 μL 1.25x NP-40 lysis buffer (1.25% NP-40, 187.5 mM NaCl, 25 mM Tris pH 7.4, 12.5 mM NaF, 1.25 mM EDTA pH 8.0, 1.25 mM ZnCl_2_ pH 4.0, 1.25 mM MgCl_2_, 1.25 mM Na_3_VO_4_, 12.5% glycerol) supplemented with aprotinin and AEBSF (Sigma). The cell debris was removed by centrifugation and the supernatant was used for determination of protein concentration (BCA Protein Assays, Thermo Fisher). Immunoprecipitations (IP) were performed consecutively with first antibodies against hEPOR (MAB 307, R&D) and JAK2 (06–255, Merck Millipore) and then with antibodies against STAT5A/B (C-17, Santa Cruz) using protein A sepharose beads. The immunoprecipitates were loaded to a 10% polyacrylamide gel and transferred to a nitrocellulose membrane (Schleicher & Schuell). The membranes were blocked with 5% BSA for 1 hour and successively incubated with a phosphotyrosine antibody (4G10, Merck Millipore). To remove antibodies, membranes were treated with β-mercaptoethanol and SDS and subsequently incubated with an anti-hEPOR antibody (C-20, Santa Cruz), an anti-JAK2 antibody (06–255, Merck Millipore) or an anti-STAT5 antibody (C-17, Santa Cruz). Secondary horseradish peroxidase-coupled antibodies were obtained from GE Healthcare or Dianova. Detection was performed using ECL substrate (GE Healthcare) and acquired with the CCD camera-based ImageQuant LAS 4000 (GE Healthcare). For quantification, the ImageQuant TL version 7.0 software (GE Healthcare) was used.

For the detection of mEPOR in mCFU-E cells, the cells were growth factor-depleted for 3 hours and 1×10^7^ cells shaking in 250 μl were stimulated for 10 min with 5 U/ml EPO alfa (Janssen-Cilag). The cells were lysed by addition of 250 μL 2x NP40 lysis buffer. IP was performed using an anti-mEPOR antibody (M-20, Santa Cruz) and protein A sepharose. For phosphorylated mEPOR, the phosphotyrosine antibody (4G10, Merck Millipore) was used. For total mEPOR anti-mEPOR antibody (M-20, Santa Cruz) was applied.

### Determination of protein abundances

For determination of protein abundances different recombinant fusion proteins were synthesized using a pGEX-2T vector (GE Healthcare). GSTΔhEPOR and GSTΔmEPOR consist of the complete cytoplasmic part of the respective receptor fused N-terminally to a GST tag leading to proteins of 52 214 Da mass for hEPOR and 52 309 Da for mEPOR. GSTΔJAK2 and GSTΔSTAT5 were constructed as described previously [61]. Briefly, in GSTΔJAK2 the tag is fused N-terminally to the kinase domain of murine JAK2 starting from L549 leading to a 94 572 Da fusion protein with 95% consensus to human JAK2. GSTΔSTAT5 consist of the N-terminal end of murine STAT5B starting from F332 which leads to a fusion protein of 78 432 Da with 97% consensus to hSTAT5B.

The concentrations of the recombinant proteins were determined using a BSA standard curve on a Coomassie-stained SDS-PAGE gel (SimplyBlue SafeStain, Invitrogen). Different amounts of the respective calibrators were added to the cell lysate. IP and immunoblotting was performed with the indicated total antibodies. The linear calibration curve based on the intensities of the recombinant protein was estimated with SigmaPlot (V12.5) and the endogenous signal was interpolated to calculate the corresponding number of molecules. The cell number was counted in parallel with a Neubauer improved counting chamber.

### Mass spectrometry

For mass spectrometry experiments, at least 1.5×10^7^ H838 or H838-HA-hEPOR cells were used per time point. Treatment, lysis and IP conditions are described above. IPs were separated by 10% SDS-PAGE and gels were stained with SimplyBlue SafeStain (Invitrogen). STAT5A (90.6 kDa) and STAT5B (89.9 kDa) containing bands were excised between around 75 and 100 kDa (according to Precision Plus Protein marker, Bio-Rad) then destained, reduced (DTT, Sigma), alkylated (IAA, Sigma) and digested with trypsin gold (Promega). To ensure robust and accurate degree of phosphorylation analysis of Tyr694 (STAT5A) and Tyr699 (STAT5B) we applied an internal standard peptide mixture (One-source peptide-/phosphopeptide ratio standard) with defined ratio of labelled peptide and its phosphorylated counterpart. Starting from the in-house synthesized stable isotope labelled phosphopeptide A-[V+6Da]-D-G-pY-V-K-P-Q-I-K (identical for STAT5A and STAT5B) the standard mixture was generated by (i) dividing peptide dilution into two aliquots (ii) quantitative dephosphorylation of one of the aliquots (iii) remixing of dephosphorylated and untreated aliquot (described in detail [62]). The standard was spiked into the samples during tryptic digestion. All mass spectrometry sample preparation steps from lysis to standard addition have been previously described in more detail [63]. Following peptide extraction and concentration of eluates, samples were desalted and purified using C18 ZipTips (Merck Millipore). Samples were measured by nanoUPLC (nanoAcquity, Waters) coupled to an LTQ-Orbitrap XL mass spectrometer (Thermo Fisher). LC separations were performed on a 75 μm × 150 mm C18 column with 1.3 μm particle size (Waters) using a water/acetonitrile based gradient up to 40% acetonitrile within 60 min. Intensities of native and labelled STAT5 peptide and phosphopeptide pairs were analyzed manually using Xcalibur 3.0.63 (Thermo Fisher).

### Analysis of the SOCS3 promoter and DNA methylation measurement

Genomatix Genome Analyzer was used for promoter analysis (www.genomatix.de). Promoter retrieval was performed by Genomatix Gene2Promoter (ElDorado 12–2013) and the transcription factor binding site analysis was performed using the Genomatix Overrepresented TFBS pipeline combining Genomatix MatBase and MatInspector based on Matrix Family Library Version 9.1. Sequence alignment was performed using the Genomatix Multiple Alignment pipeline based on DiAlign professional TF Release 3.1.5 (June 2011).

H838 and CFU-E cells were cultivated as described above. Genomic DNA was extracted with the DNeasy Tissue Kit (Qiagen) according to the manufacturer's instructions and bisulfite converted using the EZ DNA Methylation kit (Zymo Research). Amplicons spanning the entire *SOCS3* promoter region were designed and used to amplify this region from bisulfite-treated DNA (S2 Table). For MassARRAY EpiTYPER assay (Agena Bioscience), the PCR products were transcribed *in vitro*, cleaved by RNase A and subjected to matrix-assisted laser desorption ionization time-of-flight mass spectrometry to quantitatively assess methylation levels of CpG dinucleotides [64].

### Model simulation and parameter estimation

The ODE system consisted of 23 states, with 20 initial values that were implicitly dependent on kinetic parameters through steady-state assumptions, two initial values with prior knowledge available, and one initial value with prior knowledge only available for CFU-E, which was estimated for H838 & H838-HA-hEPOR. To obtain a numerical solution of the ODE system the solver CVODES was applied [65]. For CFU-E cells, 23 model variants representing experimental conditions were implemented, and 36 for H838 & H838-HA-hEPOR cells, respectively. For numerical efficiency, the ODE system and solver were compiled as C-executables, supplying calculation of states as well as sensitivities, and solved in a parallelized manner for all conditions. Data2Dynamics [31] was utilized to facilitate the automatic derivation of conditions and sensitivity equations. The ODE system was re-parameterized to disentangle internal concentrations from relative measurements and to decouple modules of the signaling network with different scales. Relative and absolute tolerances of the ODE solver were set to 10^−6^.

For parameter estimation, all three cell types CFU-E, H838 and H838-HA-hEPOR were initially implemented separately. To achieve global optimization, a multi-start deterministic optimization strategy was used [37] with 1000 initial parameter vectors for each cell type. Each single optimization was performed using the MATLAB implementation of the trust-region method (lsqnonlin) [66]. The algorithm was set to terminate an optimization run if the proposed step size is smaller than 10^−6^. To account for flat regions in the parameter space, the termination criterion based on change of the objective function was omitted. A noise parameter was assumed for each experimental technique, observable and cell type. For intensity-based measurements, a log-normal error model was used, where σ is relative to the observation [67]. For degree of phosphorylation data obtained by mass spectrometry, a constant error model was assumed. Kinetic parameters, initial concentrations, observation parameters and error model parameters were estimated simultaneously based on the data [37]. Parameters were estimated in log-space and values between 10^−5^ and 10^3^ were initially allowed. If an estimate was located at the boundary of the parameter space, the respective boundaries were enlarged. Efficiencies of inhibitors were limited to a maximum of one (100% efficiency), and the accuracy of degree of phosphorylation data obtained by mass spectrometry was restricted to a maximum of 5%.

For CFU-E cells, 516 data points were used to estimate a total of 109 parameters. 1000 parameter estimation runs were started from random points of the parameter space. The global optimum was found in 9.0% of parameter estimation runs. Likewise for H838, 204 data points were used to estimate 54 parameters. Here, the global optimum was found in 5.2%. For H838-HA-hEPOR, 407 data points were used to estimate 75 parameters with the global optimum reached in 0.7% of all optimization runs. These results suggested that the global optimum was discovered for all cell types.

### L_1_ regularization and uncertainty analysis

The comprehensive model taking all cell types into account had a total of 216 parameters that were estimated based on 1141 data points. The parameters were estimated independently for CFU-E and H838 & H838-HA-hEPOR cells, and the corresponding fold-change was L_1_-regularized. Residuals and sensitivities were passed to the optimizer. To account for discontinuities of the L_1_ regularization gradient, optimization steps were truncated in the derivatives by preventing to cross zero at each step. To facilitate convergence, derivatives of the L_1_ regularization residuals were set to zero if their contribution was smaller than the respective gradient of the log-likelihood. The re-parameterization to decouple modules of the signaling network was performed with CFU-E as reference point for H838 & H838-HA-hEPOR. For generating the regularization path, the regularization strength was scanned in log-space from 10^−4^ to 10^4^. Regularization was only used for selection of parameter differences, while for model selection the un-regularization solution was used for unbiased parameter estimates. The threshold for model selection was calculated using likelihood ratio test statistics (*α* = 0.05), i.e. by the inverse cumulative density function of the χ^2^-distribution with the difference of number of parameters between full and reduced model as degrees of freedom.

The profile likelihood was calculated to assess the standard deviation of the parameter ratios [35]. To conservatively estimate the standard deviation based on asymmetric confidence intervals, the maximum of the log_10_ difference between maximum likelihood estimate and lower/upper limit of the confidence interval was used to approximate σ.

Modeling framework, examples and data are open source and publicly available at www.data2dynamics.org.

## Supporting Information

S1 Table Cell volume and protein abundance of H838, H838-HA-hEPOR and CFU-E cells. For H838 cells, the cytoplasmic and nuclear volumes were derived as shown in S3 Fig. The amount of total EPOR per H838-HA-hEPOR cell and per CFU-E cell was quantified as shown in S1D Fig. The number of EPOR molecules per H838 cell was calculated by taking the relative EPOR ratio of those cells shown in S1B Fig into account. The number of JAK2 molecules per H838 cell was calculated as shown in S2C Fig and number of STAT5 molecules per H838 cell was calculated as shown in S2D Fig. For the mathematical model, the amounts of the EPOR-JAK2 complex and of STAT5 were converted to molar concentrations. * CFU-E data that was previously published [19]. ** A percentage of 20% as previously suggested for CFU-E cells [19] was assumed for the calculation of the amount of EPOR on the cell surface in H838 cells. *** It was shown that the amount of pEPOR on the cell surface of H838-HA-hEPOR cells is approximately 12 fold higher compared to H838 cells (S1D Fig). This number was used to extrapolate the amount of EPOR on the cell surface of H838-HA-hEPOR cells.
(DOCX)

S2 Table Primers utilized to amplify the SOCS3 promoter region in CFU-E and H838 cells for DNA methylation measurement. Primer pairs to obtain *SOCS3* promoter amplicons are indicated (F: forward, R: reverse). Bases indicated with upper case letters denote DNA binding sequences. Lower case letters indicate tag sequences used for MassARRAY EpiTYPER assay (T7 promoter sequences and random sequences, respectively).
(DOCX)

S1 Fig Quantification of the EPOR in the NSCLC cell line H838 and its derivative H838-HA-EPOR and mouse CFU-E cells. (A) The immunoblot of total EPOR from Fig 1A is shown with different exposure times to display both, low and high EPOR signals. The relative amounts of EPOR were quantified for H838 and H838-HA-hEPOR cells. (B) The amount of the total EPOR protein of H838-HA-hEPOR cells is shown relative to the amount of EPOR in H838 cells. (C) The abundance of phosphorylated EPOR protein of EPO-stimulated H838-HA-hEPOR cells is shown relative to the abundance of EPO-stimulated pEPOR of H838 cells. (D) For absolute quantification of the EPOR, H838-HA-hEPOR and CFU-E cells were lysed. The lysate of 8 280 000 CFU-E cells was added to the 100 ng sample of a murine EPOR calibrator (GST-ΔmEPOR) dilution series and the lysate of 228 000 H838-HA-hEPOR cells was added to the 3 ng sample of a human EPOR calibrator (GST-ΔhEPOR) dilution series. EPOR was subjected to immunoprecipitation (IP) and quantitative immunoblotting (IB). One representative immunoblot out of a biological triplicate is shown. The amount of EPOR per cell was calculated with a cell-specific calibration curve based on all replicates.
(PDF)

S2 Fig Comparison of EPO alfa and EPO beta in H838-HA-hEPOR cells and quantification of JAK2 and STAT5 in H838 cells. (A) H838-HA-hEPOR cells were either stimulated with 10 U/ml EPO alfa (black) or 10 U/ml EPO beta (red). The cells were lysed after 10 min and hEPOR and JAK2 proteins were subjected to immunoprecipitation (IP) and phosphorylated EPOR and JAK2 were detected by quantitative immunoblotting (IB). The experiment was performed in two independent replicates. (B) The measured data in (A) is depicted as black (EPO alfa) or red (EPO beta) closed circles and estimated by a phenomenological mathematical model (black and red lines). Shading represents estimated experimental error. (C) The lysate of 5×10^6^ H838 cells each was added to a dilution series of JAK2 calibrator (GST-ΔJAK2). JAK2 was subjected to IP and IB. One representative immunoblot out of biological triplicates is shown. The amount of JAK2 was calculated with a calibration curve based on all replicates. (D) The lysate of 5×10^6^ H838 cells each was added to a dilution series of STAT5 calibrator (GST-ΔSTAT5). STAT5 was subjected to IP and IB. One representative immunoblot out of biological triplicates is shown. The amount of STAT5 was calculated with a calibration curve based on all replicates.
(PDF)

S3 Fig Determination of the cellular and nuclear diameters of H838 cells. (A) H838 cells expressing GFP (green) were trypsinized and nuclei were stained with Hoechst (blue). Confocal images were acquired and the diameters of the nuclei (D_nucleus_) and the cell (D_cell_) were determined. The results are summarized in S1 Table. One exemplary image is shown. Scale bar: 20 μm. (B) Distribution of the cellular and nuclear diameters of H838 cells is shown (n = 206).
(PDF)

S4 Fig Increased viability of cisplatin-treated H838 and H838-HA-hEPOR cells upon co-treatment with EPO beta. H838 (A) cells or H838-HA-hEPOR cells (B) were treated for three days with 5 mg/l cisplatin or left untreated. Additionally, cells were treated with or without 10 U/ml EPO beta and the cell viability was measured with CellTiter-Blue assay. The error bars represent standard deviation of biological replicates (n ≥ 5). The assay was performed in two independent experiments (first replicate is shown in Fig 1D).
(PDF)

S5 Fig Reduced apoptosis of cisplatin-treated H838-HA-hEPOR cells upon co-treatment with EPO beta. H838-HA-hEPOR cells expressing the Casper3-GR FRET-based sensor (H838-HA-hEPOR-Casper3-GR) were treated with 5 mg/l cisplatin, 10 U/ml EPO beta, a combination of both or left untreated. Casper3-GR FRET signal was measured by life-cell imaging for 65 hours. Caspase-3 activity was determined based on the green-to-red ratio and normalized to the untreated control (n = 2, first replicate is shown in Fig 1E).
(PDF)

S6 Fig Regularization path and parameter differences of the two-step phosphorylation reaction example. (A) The regularization path of parameter differences for the model and the simulated data depicted in Fig 2 is shown. At the regularization weight corresponding to the parsimonious model (*λ* ≈ 60), *k*_3_ and [Protein]_t = 0_ were identified as cell type-specific parameters (indicated with asterisks). (B) The profile likelihood approach was used to determine the confidence interval of the parameter differences identified in (A). The parameter differences (log_10_ fold-changes) are not compatible with zero, validating the result of the algorithm.
(PDF)

S7 Fig Quantitative data measured in CFU-E, H838 and H838-HA-hEPOR cells. (A) Quantitative measurement of mRNAs by quantitative qRT-PCR in CFU-E cells. (B) Quantitative measurement of mRNAs and proteins by quantitative qRT-PCR, quantitative immunoblotting and mass spectrometry in H838-HA-hEPOR cells. (C) Quantitative measurement of the EPO dose-dependency of pEPOR and pJAK2 by quantitative immunoblotting in H838 and H838-HA-hEPOR cells. (D) Quantitative measurement of mRNAs and proteins by quantitative qRT-PCR, quantitative immunoblotting and mass spectrometry in H838 cells.
(PDF)

S8 Fig Trajectories of parsimonious model based on CFU-E data. Experimental CFU-E data are shown with open and closed circles, trajectories of the parsimonious model are depicted with solid lines. Shading represents estimated experimental error.
(PDF)

S9 Fig Trajectories of parsimonious model based on H838 and H838-HA-hEPOR data. (A) Experimental H838-HA-hEPOR data are shown with closed circles, trajectories of the parsimonious model are depicted with solid lines. Shading represents estimated experimental error. (B) Experimental H838 and H838-HA-hEPOR data are shown with closed circles, trajectories of the parsimonious model are depicted with solid lines. Shading represents estimated experimental error. (C) Experimental H838 data are shown with closed circles, trajectories of the parsimonious model are depicted with solid lines. Shading represents estimated experimental error.
(PDF)

S10 Fig Regularization path and parameter differences of the generalized mathematical model structure of the EPO-induced JAK2/STAT5 signaling pathway. (A) The regularization path of parameter differences is shown for the generalized model structure as displayed in Fig 4, and the experimental data in CFU-E, H838 and H838-HA-hEPOR cells as displayed in S7 Fig. The regularization paths are not necessarily monotonously drifting towards zero with increasing regularization weight *λ*. The asterisks depict the identified parameter differences. (B) The profile likelihood approach was used to determine the confidence interval of the parameter differences identified in (A). All identified parameter differences (log_10_ fold-changes) are not compatible with zero, validating the result of the algorithm.
(PDF)

S11 Fig Differential behavior of the *SOCS3* promotor in CFU-E and H838 cells. (A) pSTAT5 data and *SOCS3* mRNA data are shown with the parsimonious model trajectories in CFU-E cells. Shading represents estimated experimental error. The *SOCS3* promoter activity in the relevant range of npSTAT5 was calculated. (B) pSTAT5 data and *SOCS3* mRNA data are shown with the parsimonious model trajectory in H838 & H838-HA-hEPOR cells. Shading represents estimated experimental error. *SOCS3* promoter activity in the relevant range of npSTAT5 was calculated. (C) The *SOCS3* promoter was compared between *Mus musculus* and *Homo sapiens*. TSS: transcription start site of *SOCS3*. Strand direction is indicated relative to the *SOCS3* coding sequence. Asterisks indicate conserved bases between mouse and human. The transcription factor binding site matches are displayed as green boxes within the alignment. Bases in capital letters denote the core sequence used and red bases indicate that the matrix exhibits a high conservation at this position. Methylation of CpG islands measured by MassARRAY in CFU-E and H838 cells is indicated.
(PDF)

S12 Fig Sensitivity analysis of the parsimonious model for CFU-E and H838 cells. Control coefficients determined for the 25 kinetic parameters in CFU-E and H838 cells are shown. The area-under-curve of npSTAT5 at 60 min after stimulation was used as read-out to calculate the sensitivities. Asterisks indicate parameters exerting more control in H838 compared to CFU-E cells.
(PDF)
