# Supplementary material for: Identification of Cell Type-Specific Differences in Erythropoietin Receptor Signaling in Primary Erythroid and Lung Cancer Cells
Source: PLoS Comput Biol. 2016 Aug 5;12(8):e1005049. doi: 10.1371/journal.pcbi.1005049 (PMC4975441; doi:10.1371/journal.pcbi.1005049)
Supplement: S1 Table — For H838 cells, the cytoplasmic and nuclear volumes were derived as shown in S3 Fig. The amount of total EPOR per H838-HA-hEPOR cell and per CFU-E cell was quantified as shown in S1D Fig. The number of EPOR molecules per H838 cell was calculated by taking the relative EPOR ratio of those cells shown in S1B Fig into account. The number of JAK2 molecules per H838 cell was calculated as shown in S2C Fig and number of STAT5 molecules per H838 cell was calculated as shown in S2D Fig. For the mathematical model, the amounts of the EPOR-JAK2 complex and of STAT5 were converted to molar concentrations. * CFU-E data that was previously published [19]. ** A percentage of 20% as previously suggested for CFU-E cells [19] was assumed for the calculation of the amount of EPOR on the cell surface in H838 cells. *** It was shown that the amount of pEPOR on the cell surface of H838-HA-hEPOR cells is approximately 12 fold higher compared to H838 cells (S1D Fig). This number was used to extrapolate the amount of EPOR on the cell surface of H838-HA-hEPOR cells. (DOCX) [file pcbi.1005049.s001.docx]

**Merkle, Steiert et al., S1 Table**

| **Measured concentrations** | | | | |
| --- | --- | --- | --- | --- |
|  |  | **CFU-E** | **H838** | **H838-HA-hEPOR** |
| Volume (µm^3^) | Cell | 675 * | 13 718 ± 8 266 | |
|  | Nucleus | 275 * | 2 154 ± 1 225 | |
|  | Cytoplasm | 400 * | 11 564 ± 7 468 | |
| Molecules/cell | Total EPOR | 4 300 ± 2 200 | 3 600 ± 1 200 | 620 000 ± 200 000 |
|  | EPOR on cell surface | 1 000 * | 837 ** | 10 034 *** |
|  | JAK2 | 23 700 * | 1 174 ± 421 | |
|  | STAT5 | 20 000 * | 90 163 ± 24 930 | |
|  | | | | |
| **Calculated concentrations used for mathematical modeling** | | | | |
|  |  | **CFU-E** | **H838** | **H838-HA-hEPOR** |
| Initial concentrations (nM) | EPOR-JAK2 complex | 4.15 | 0.12 | 1.44 |
|  | STAT5 | 83.03 | 12.95 | |
