# Supplementary material for: Identification of Cell Type-Specific Differences in Erythropoietin Receptor Signaling in Primary Erythroid and Lung Cancer Cells
Source: PLoS Comput Biol. 2016 Aug 5;12(8):e1005049. doi: 10.1371/journal.pcbi.1005049 (PMC4975441; doi:10.1371/journal.pcbi.1005049)
Supplement: S2 Table — Primer pairs to obtain SOCS3 promoter amplicons are indicated (F: forward, R: reverse). Bases indicated with upper case letters denote DNA binding sequences. Lower case letters indicate tag sequences used for MassARRAY EpiTYPER assay (T7 promoter sequences and random sequences, respectively). (DOCX) [file pcbi.1005049.s002.docx]

**Merkle, Steiert et al., S2 Table**

| ***Mus musculus*** |  |
| --- | --- |
| **Description** | **Sequence** |
| mSOCS3_F1 | aggaagagagGTTATTATTTTGTATTGAAAGGTTGTG |
| mSOCS3_R1 | cagtaataCGactcactatagggagaaggctCACTTCCTAAATCCCCAATAAACTA |
| mSOCS3_F2 | aggaagagagTAGTTTATTGGGGATTTAGGAAGTG |
| mSOCS3_R2 | cagtaataCGactcactatagggagaaggctTTCCTTCTCAAATTTAACCCTATCC |
| mSOCS3_F3 | aggaagagagAGGATAGGGTTAAATTTGAGAAGGA |
| mSOCS3_R3 | cagtaatacgactcactatagggagaaggctCAAAAAACCCACTAAAAACAAACAC |
|  |  |
| ***Homo sapiens*** |  |
| **Description** | **Sequence** |
| hSOCS3_F1 | aggaagagagGGGAGGGGATTAGGAGAGGGAT |
| hSOCS3_R1 | cagtaatacgactcactatagggagaaggctCCTCTACCAAAAATCAACCTTCTTA |
| hSOCS3_F2 | aggaagagagTTTTTTTAAGAAGGTTGATTTTTGG |
| hSOCS3_R2 | cagtaatacgactcactatagggagaaggctCCAAATTTACAAACCCCAATACATA |
| hSOCS3_F3 | aggaagagagTATGTATTGGGGTTTGTAAATTTGG |
| hSOCS3_R3 | cagtaatacgactcactatagggagaaggctAAAACAAAAAATCACATTCCAAAAA |
| hSOCS3_F4 | aggaagagagTATTTTAGGTTGGGGTTAGATTTGG |
| hSOCS3_R4 | cagtaatacgactcactatagggagaaggctCACACAACCCATTTAAAAACAAAA |
