# Supplementary figures and images for: Identification of Cell Type-Specific Differences in Erythropoietin Receptor Signaling in Primary Erythroid and Lung Cancer Cells
Source: PLoS Comput Biol. 2016 Aug 5;12(8):e1005049. doi: 10.1371/journal.pcbi.1005049 (PMC4975441; doi:10.1371/journal.pcbi.1005049)

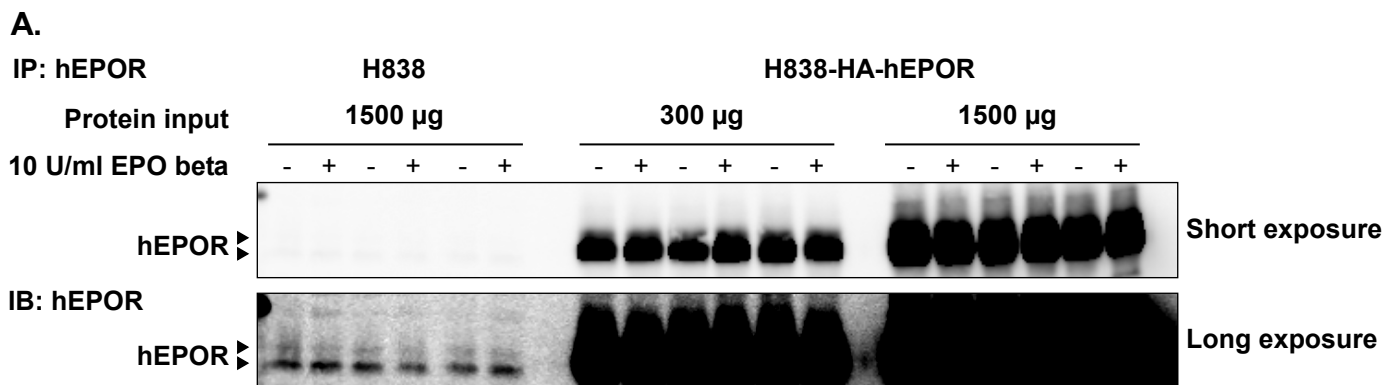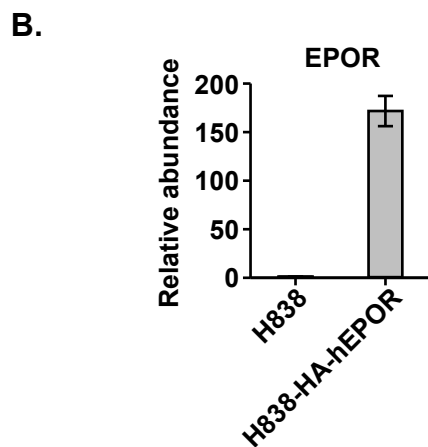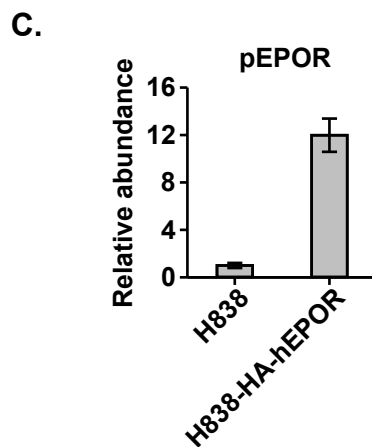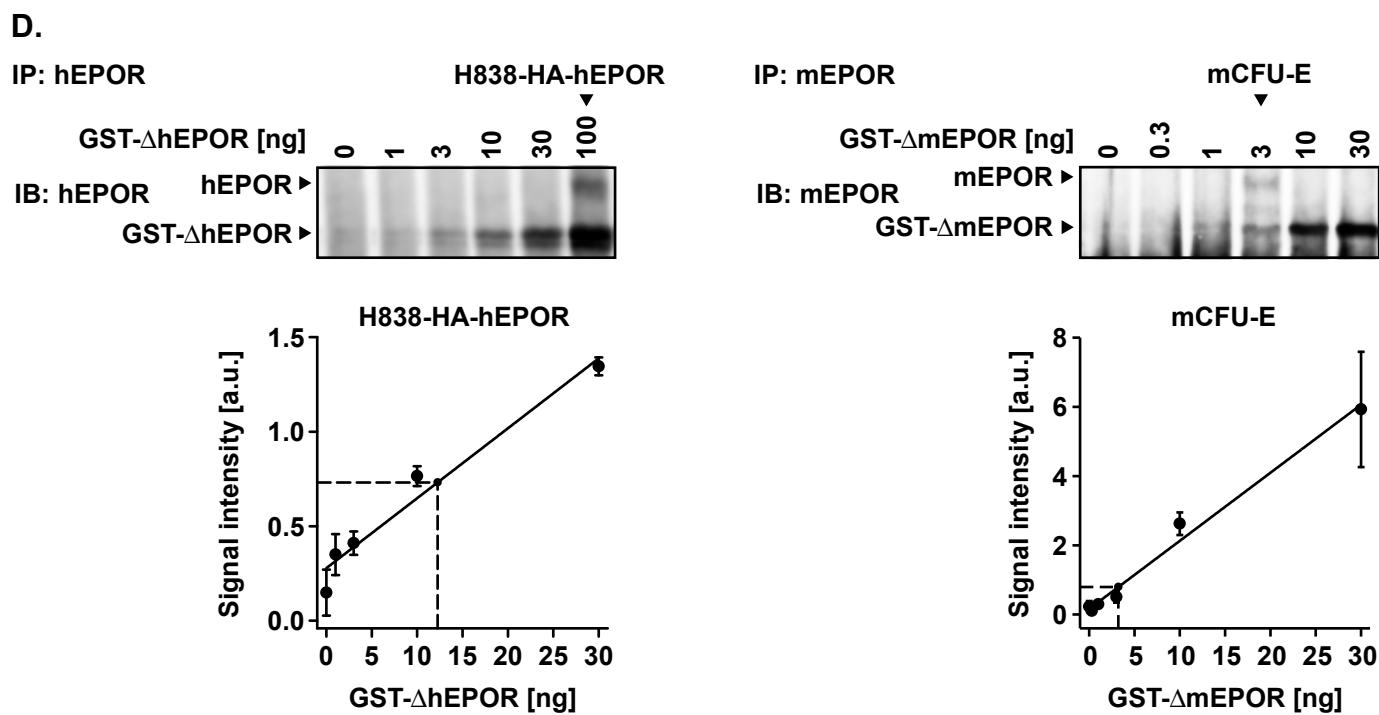

Merkle, Steiert et al., S1 Figure

Supplement: S1 Fig — (A) The immunoblot of total EPOR from Fig 1A is shown with different exposure times to display both, low and high EPOR signals. The relative amounts of EPOR were quantified for H838 and H838-HA-hEPOR cells. (B) The amount of the total EPOR protein of H838-HA-hEPOR cells is shown relative to the amount of EPOR in H838 cells. (C) The abundance of phosphorylated EPOR protein of EPO-stimulated H838-HA-hEPOR cells is shown relative to the abundance of EPO-stimulated pEPOR of H838 cells. (D) For absolute quantification of the EPOR, H838-HA-hEPOR and CFU-E cells were lysed. The lysate of 8 280 000 CFU-E cells was added to the 100 ng sample of a murine EPOR calibrator (GST-ΔmEPOR) dilution series and the lysate of 228 000 H838-HA-hEPOR cells was added to the 3 ng sample of a human EPOR calibrator (GST-ΔhEPOR) dilution series. EPOR was subjected to immunoprecipitation (IP) and quantitative immunoblotting (IB). One representative immunoblot out of a biological triplicate is shown. The amount of EPOR per cell was calculated with a cell-specific calibration curve based on all replicates. (PDF) [file pcbi.1005049.s003.pdf]

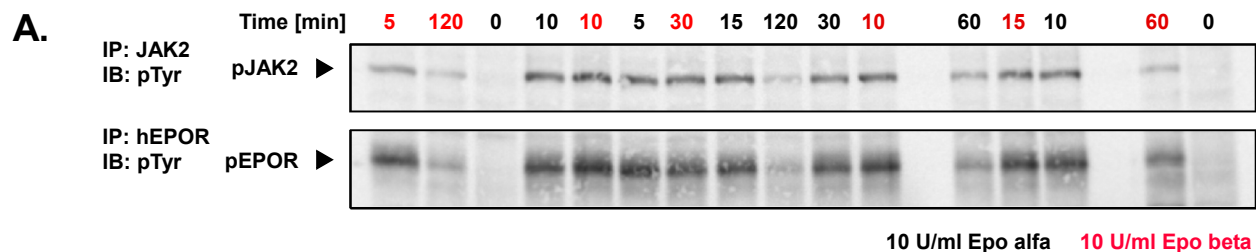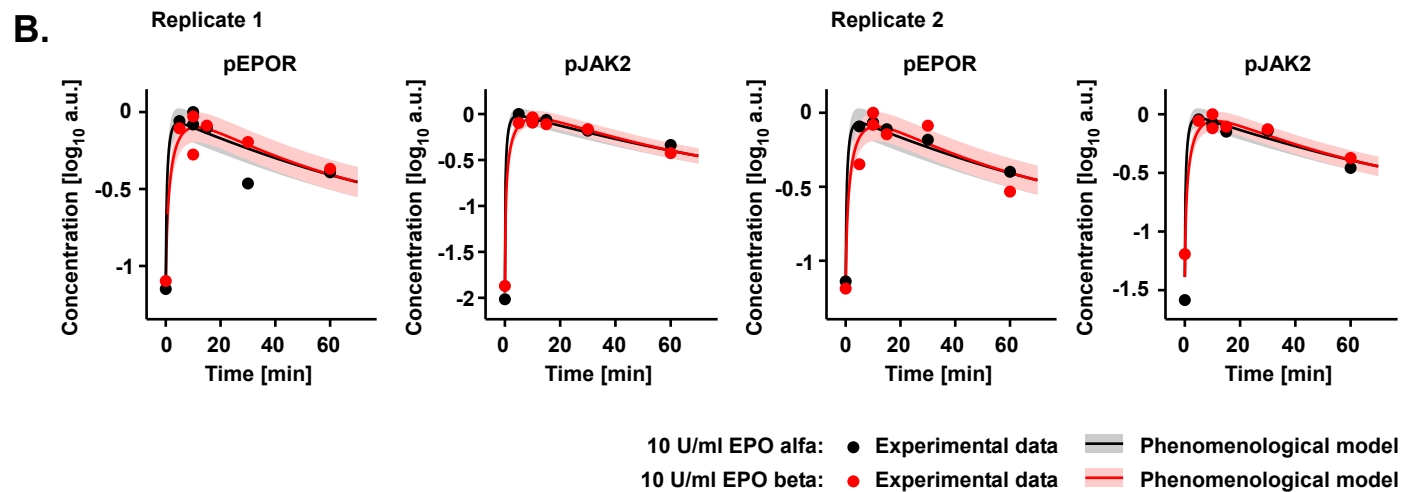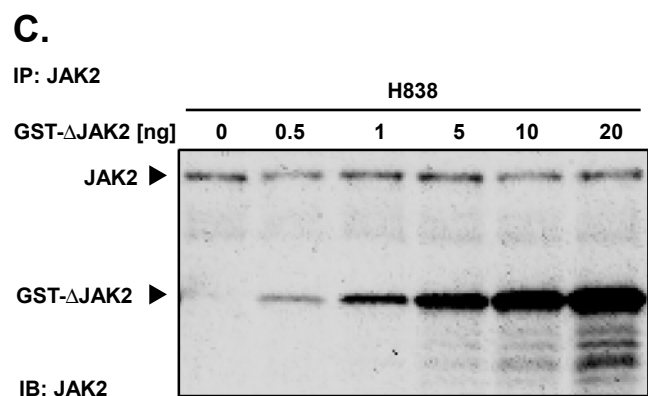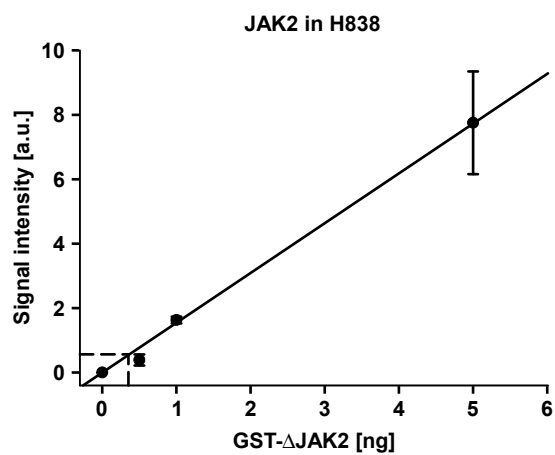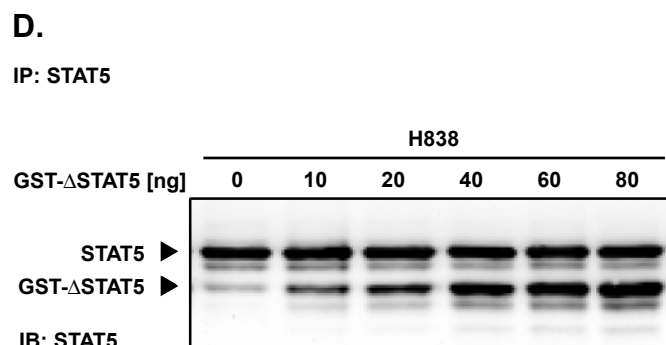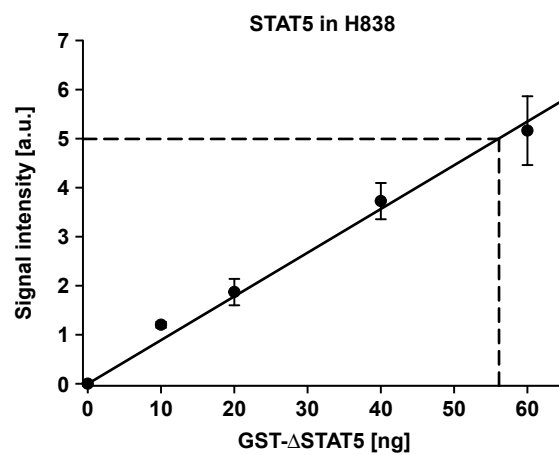

Supplement: S2 Fig — (A) H838-HA-hEPOR cells were either stimulated with 10 U/ml EPO alfa (black) or 10 U/ml EPO beta (red). The cells were lysed after 10 min and hEPOR and JAK2 proteins were subjected to immunoprecipitation (IP) and phosphorylated EPOR and JAK2 were detected by quantitative immunoblotting (IB). The experiment was performed in two independent replicates. (B) The measured data in (A) is depicted as black (EPO alfa) or red (EPO beta) closed circles and estimated by a phenomenological mathematical model (black and red lines). Shading represents estimated experimental error. (C) The lysate of 5×10^6^ H838 cells each was added to a dilution series of JAK2 calibrator (GST-ΔJAK2). JAK2 was subjected to IP and IB. One representative immunoblot out of biological triplicates is shown. The amount of JAK2 was calculated with a calibration curve based on all replicates. (D) The lysate of 5×10^6^ H838 cells each was added to a dilution series of STAT5 calibrator (GST-ΔSTAT5). STAT5 was subjected to IP and IB. One representative immunoblot out of biological triplicates is shown. The amount of STAT5 was calculated with a calibration curve based on all replicates. (PDF) [file pcbi.1005049.s004.pdf]

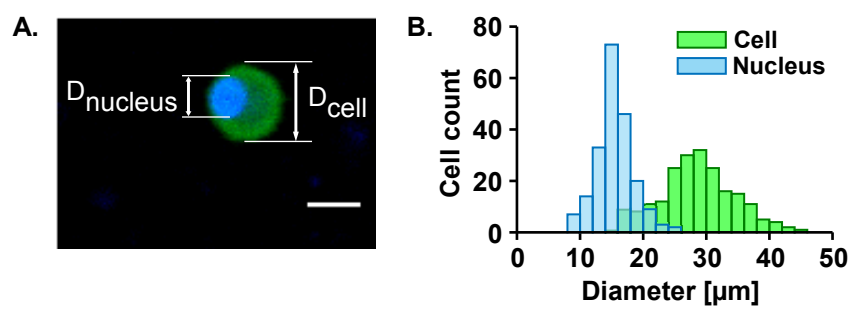

Merkle, Steiert et al., S3 Figure

Supplement: S3 Fig — (A) H838 cells expressing GFP (green) were trypsinized and nuclei were stained with Hoechst (blue). Confocal images were acquired and the diameters of the nuclei (Dnucleus) and the cell (Dcell) were determined. The results are summarized in S1 Table. One exemplary image is shown. Scale bar: 20 μm. (B) Distribution of the cellular and nuclear diameters of H838 cells is shown (n = 206). (PDF) [file pcbi.1005049.s005.pdf]

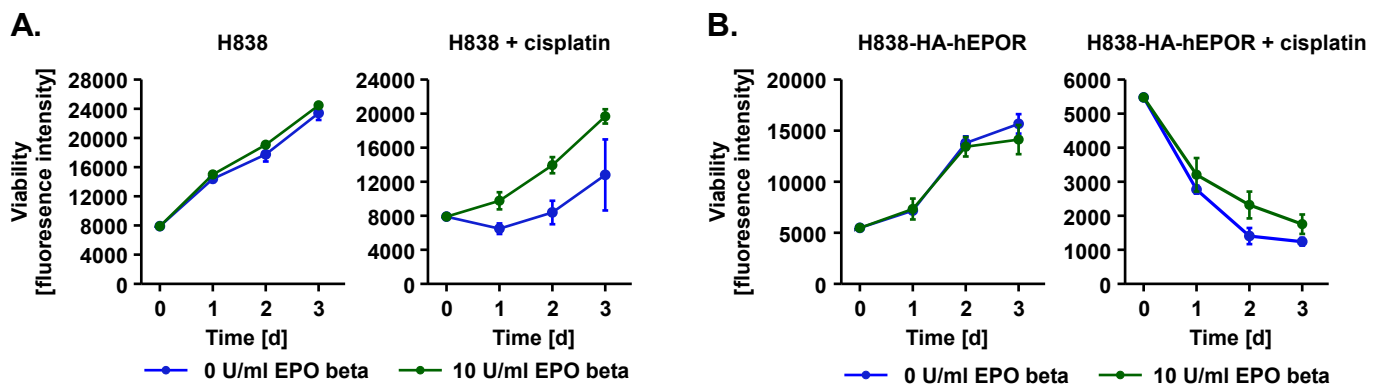

Merkle, Steiert et al., S4 Figure

Supplement: S4 Fig — H838 (A) cells or H838-HA-hEPOR cells (B) were treated for three days with 5 mg/l cisplatin or left untreated. Additionally, cells were treated with or without 10 U/ml EPO beta and the cell viability was measured with CellTiter-Blue assay. The error bars represent standard deviation of biological replicates (n ≥ 5). The assay was performed in two independent experiments (first replicate is shown in Fig 1D). (PDF) [file pcbi.1005049.s006.pdf]

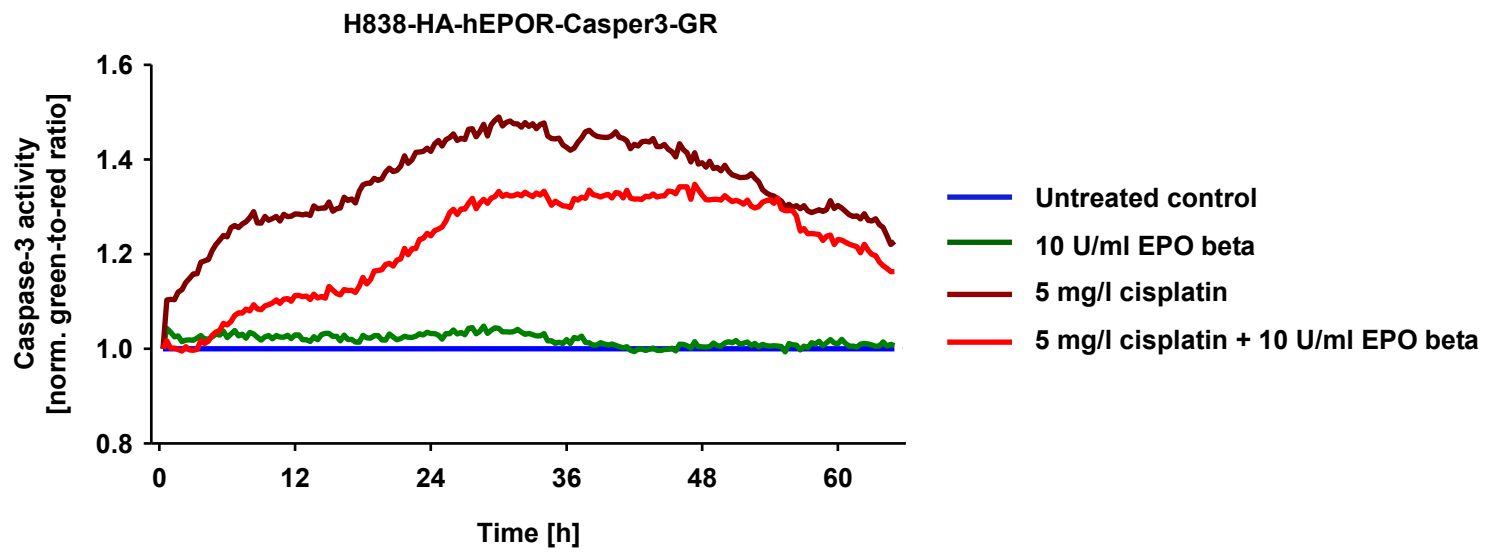

Merkle, Steiert et al., S5 Figure

Supplement: S5 Fig — H838-HA-hEPOR cells expressing the Casper3-GR FRET-based sensor (H838-HA-hEPOR-Casper3-GR) were treated with 5 mg/l cisplatin, 10 U/ml EPO beta, a combination of both or left untreated. Casper3-GR FRET signal was measured by life-cell imaging for 65 hours. Caspase-3 activity was determined based on the green-to-red ratio and normalized to the untreated control (n = 2, first replicate is shown in Fig 1E). (PDF) [file pcbi.1005049.s007.pdf]

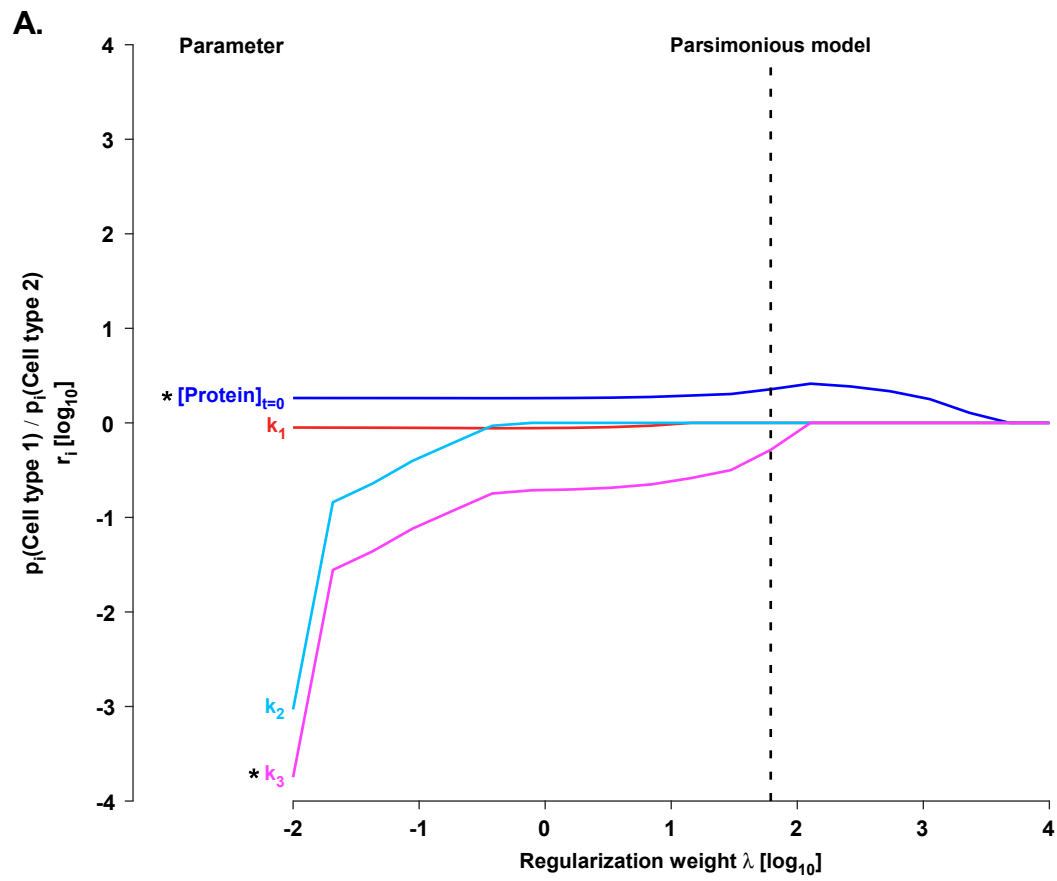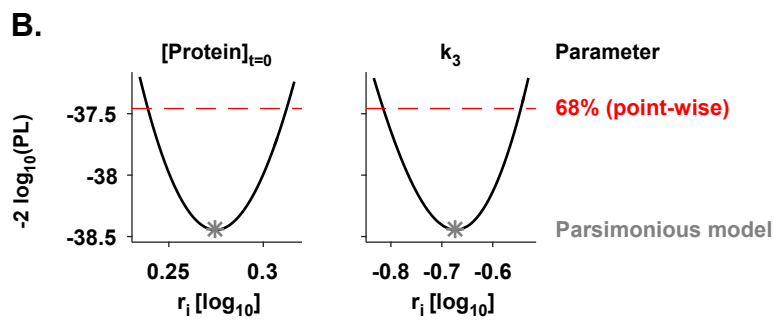

Merkle, Steiert et al., S6 Figure

Supplement: S6 Fig — (A) The regularization path of parameter differences for the model and the simulated data depicted in Fig 2 is shown. At the regularization weight corresponding to the parsimonious model (λ ≈ 60), k3 and [Protein]t = 0 were identified as cell type-specific parameters (indicated with asterisks). (B) The profile likelihood approach was used to determine the confidence interval of the parameter differences identified in (A). The parameter differences (log10 fold-changes) are not compatible with zero, validating the result of the algorithm. (PDF) [file pcbi.1005049.s008.pdf]

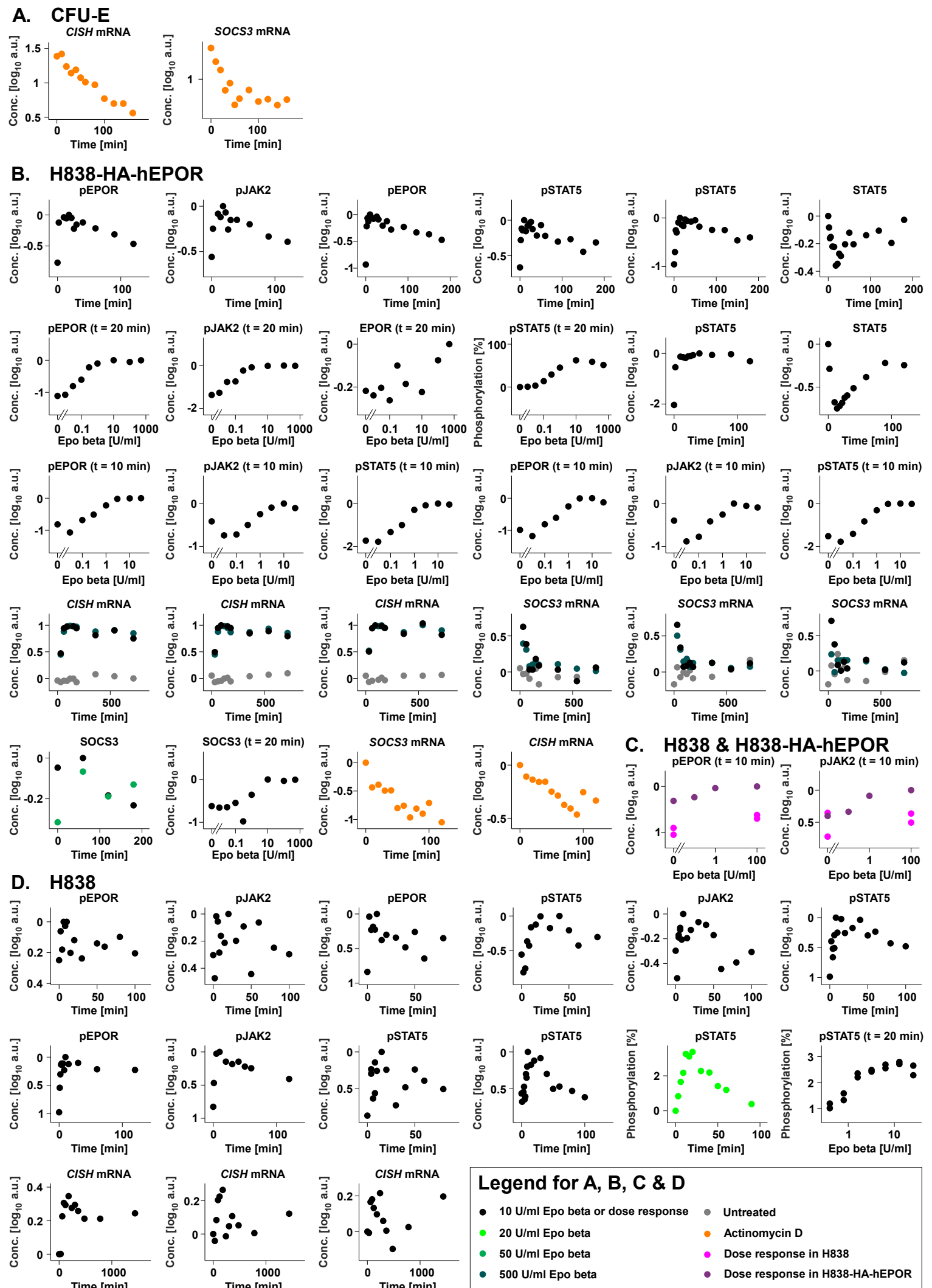

Supplement: S7 Fig — (A) Quantitative measurement of mRNAs by quantitative qRT-PCR in CFU-E cells. (B) Quantitative measurement of mRNAs and proteins by quantitative qRT-PCR, quantitative immunoblotting and mass spectrometry in H838-HA-hEPOR cells. (C) Quantitative measurement of the EPO dose-dependency of pEPOR and pJAK2 by quantitative immunoblotting in H838 and H838-HA-hEPOR cells. (D) Quantitative measurement of mRNAs and proteins by quantitative qRT-PCR, quantitative immunoblotting and mass spectrometry in H838 cells. (PDF) [file pcbi.1005049.s009.pdf]

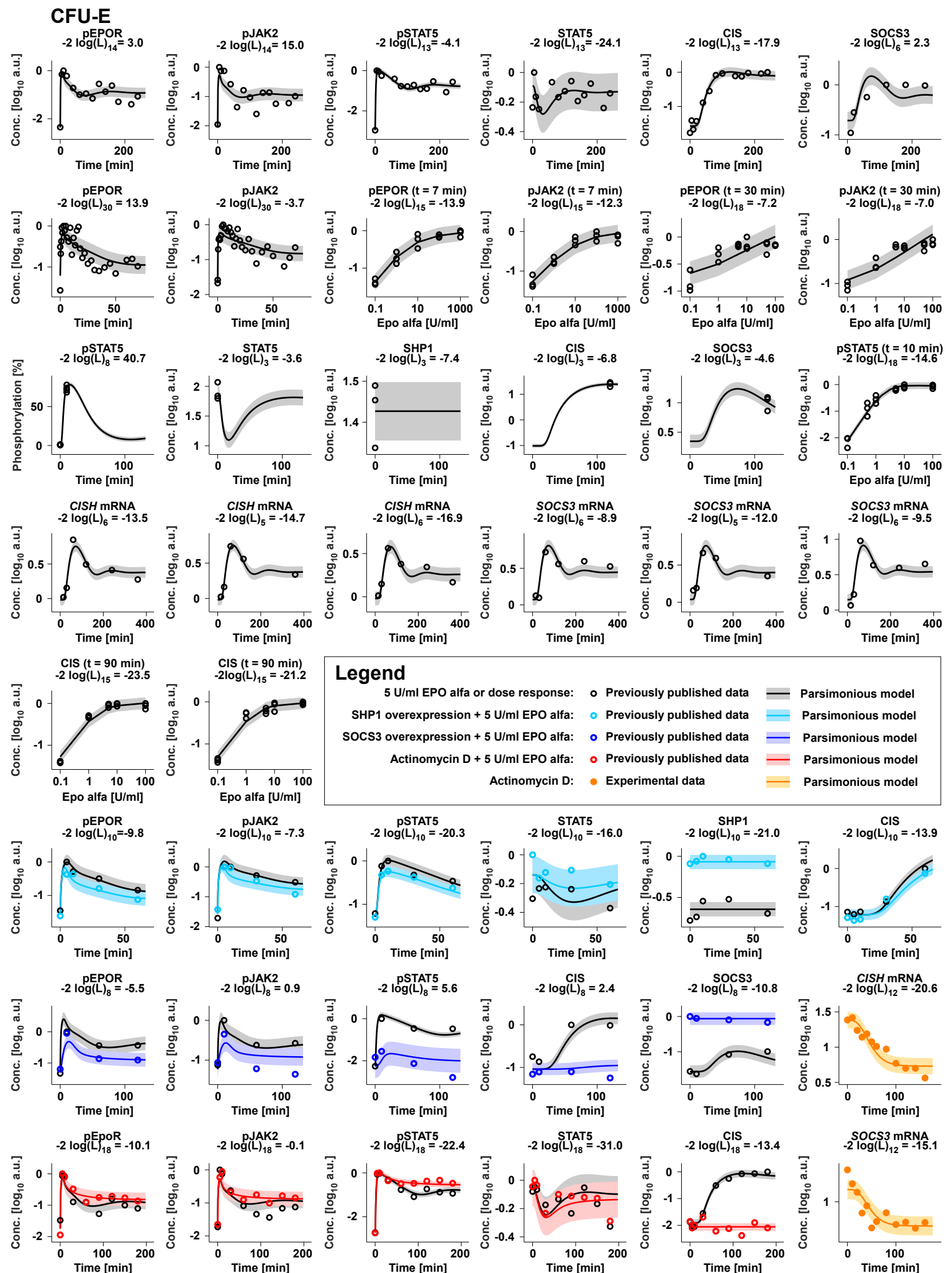

Supplement: S8 Fig — Experimental CFU-E data are shown with open and closed circles, trajectories of the parsimonious model are depicted with solid lines. Shading represents estimated experimental error. (PDF) [file pcbi.1005049.s010.pdf]

### A. H838-HA-hEPOR

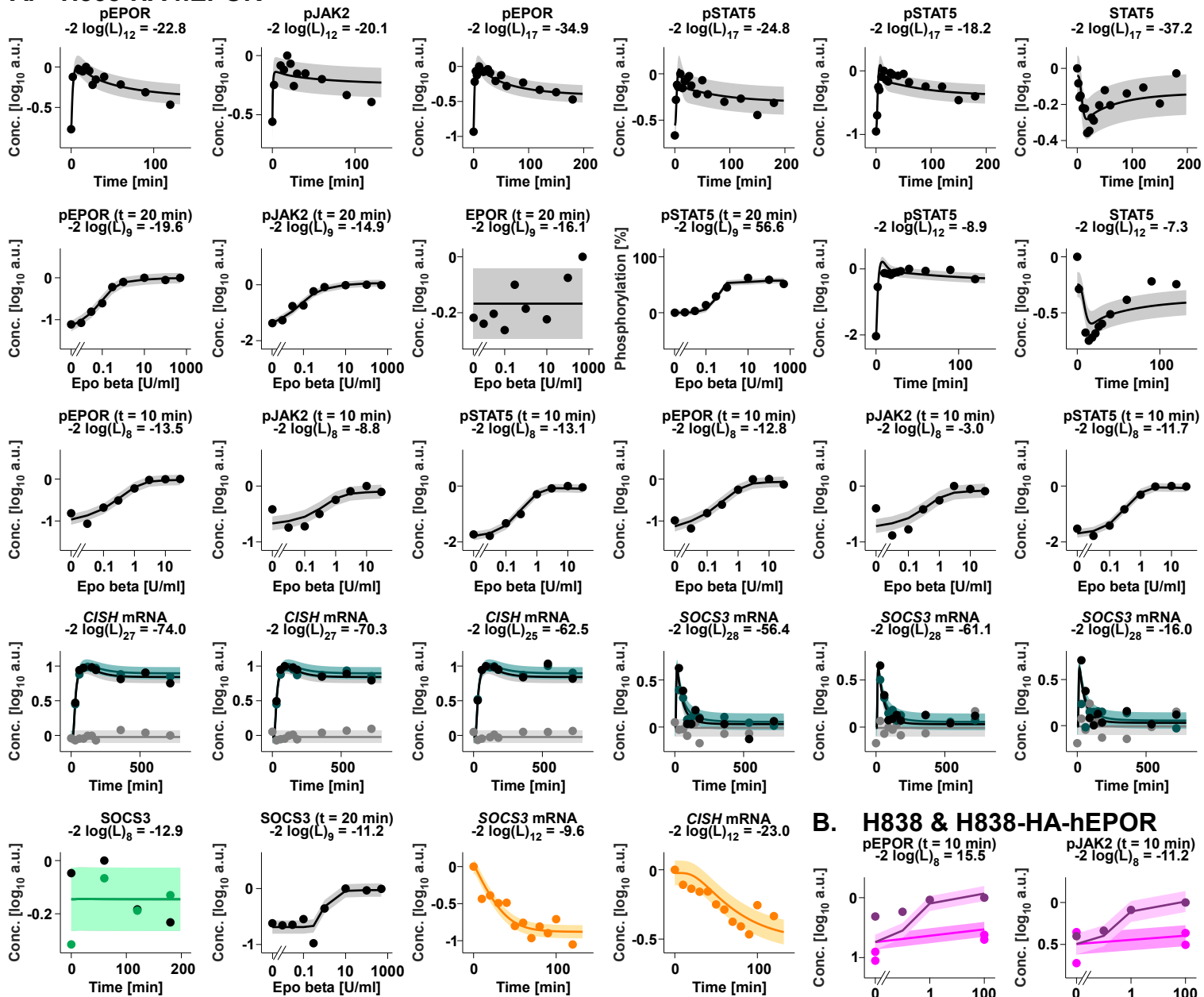

### C. H838

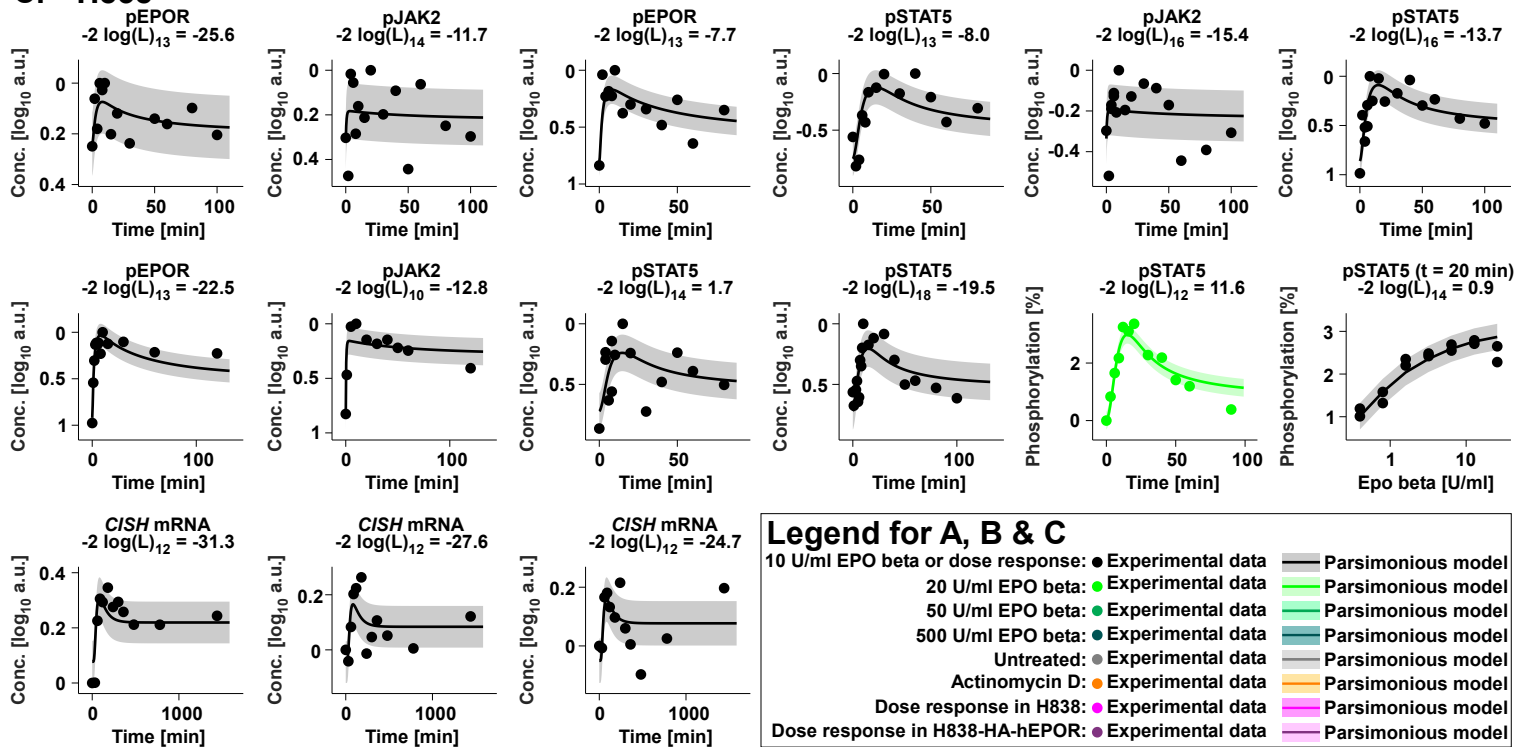

Merkle, Steiert et al., S9 Figure

Supplement: S9 Fig — (A) Experimental H838-HA-hEPOR data are shown with closed circles, trajectories of the parsimonious model are depicted with solid lines. Shading represents estimated experimental error. (B) Experimental H838 and H838-HA-hEPOR data are shown with closed circles, trajectories of the parsimonious model are depicted with solid lines. Shading represents estimated experimental error. (C) Experimental H838 data are shown with closed circles, trajectories of the parsimonious model are depicted with solid lines. Shading represents estimated experimental error. (PDF) [file pcbi.1005049.s011.pdf]

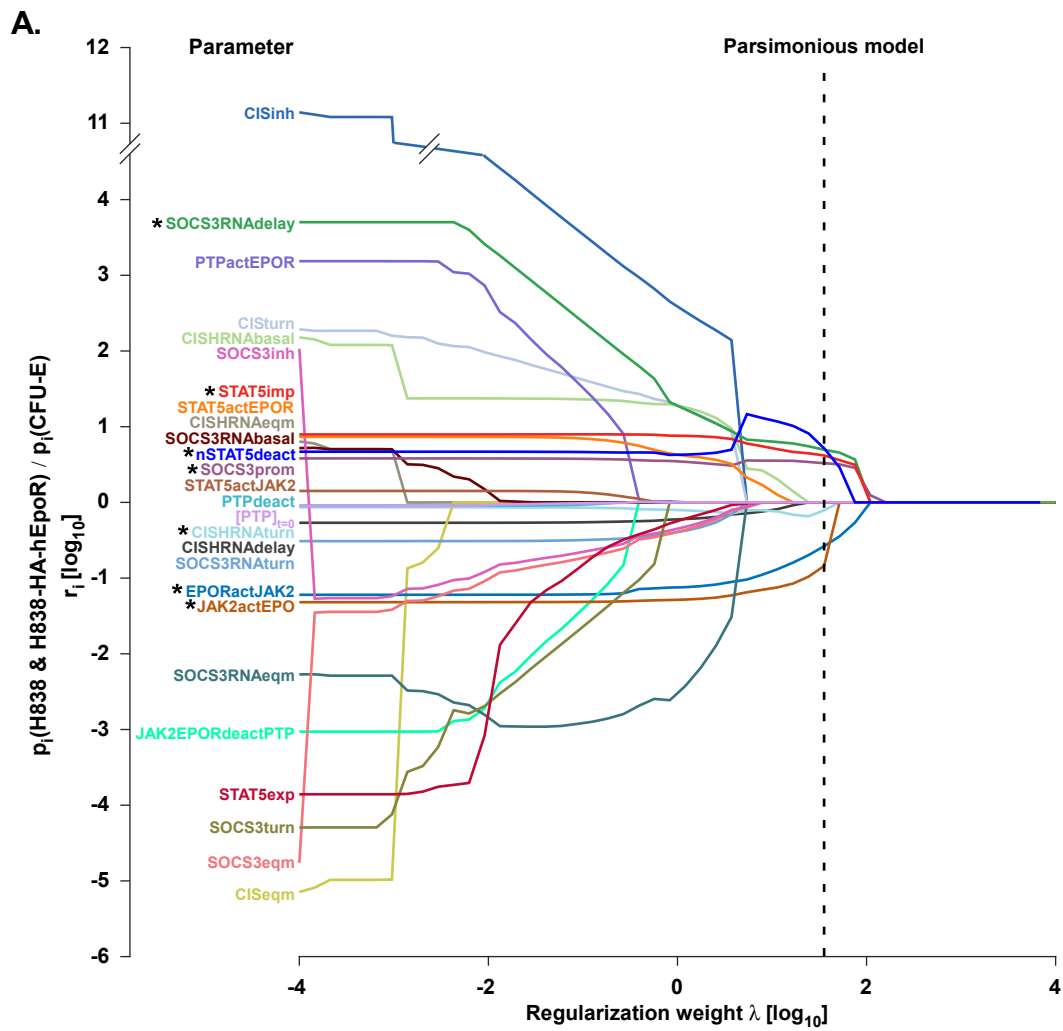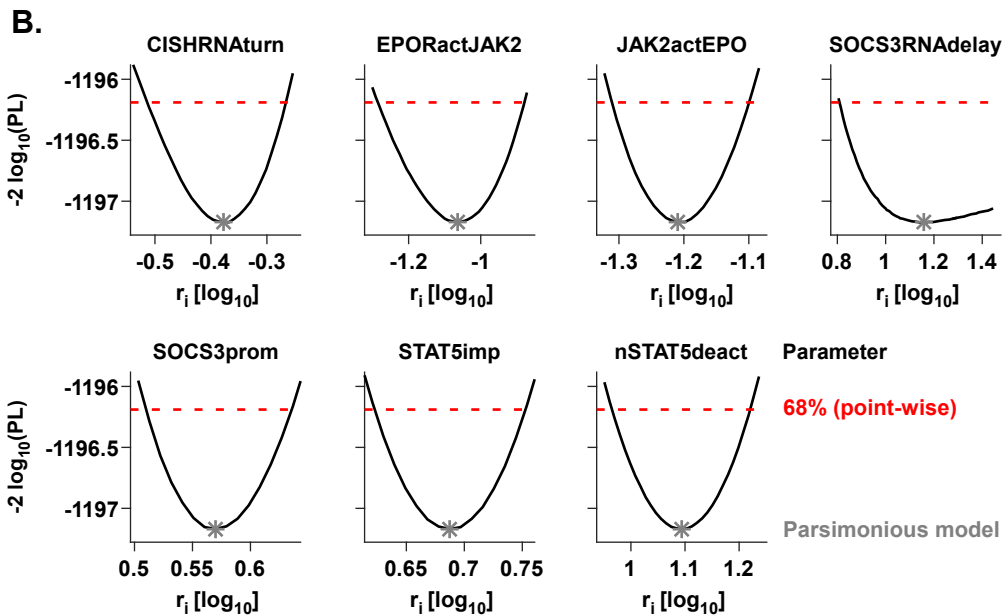

Merkle, Steiert et al., S10 Figure

Supplement: S10 Fig — (A) The regularization path of parameter differences is shown for the generalized model structure as displayed in Fig 4, and the experimental data in CFU-E, H838 and H838-HA-hEPOR cells as displayed in S7 Fig. The regularization paths are not necessarily monotonously drifting towards zero with increasing regularization weight λ. The asterisks depict the identified parameter differences. (B) The profile likelihood approach was used to determine the confidence interval of the parameter differences identified in (A). All identified parameter differences (log10 fold-changes) are not compatible with zero, validating the result of the algorithm. (PDF) [file pcbi.1005049.s012.pdf]

A. CFU-E

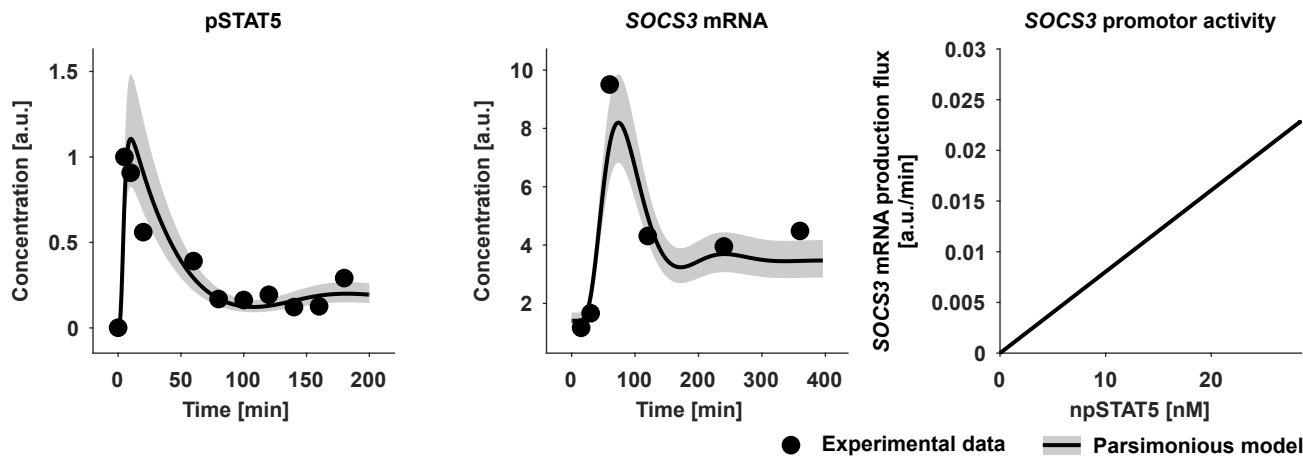

B. H838 & H838-HA-HEPOR

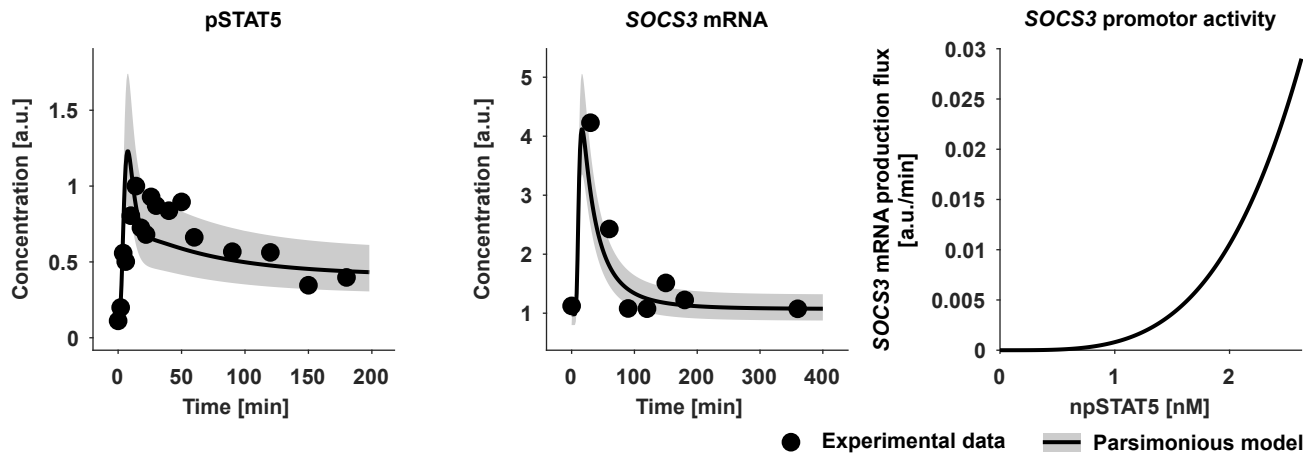

C.

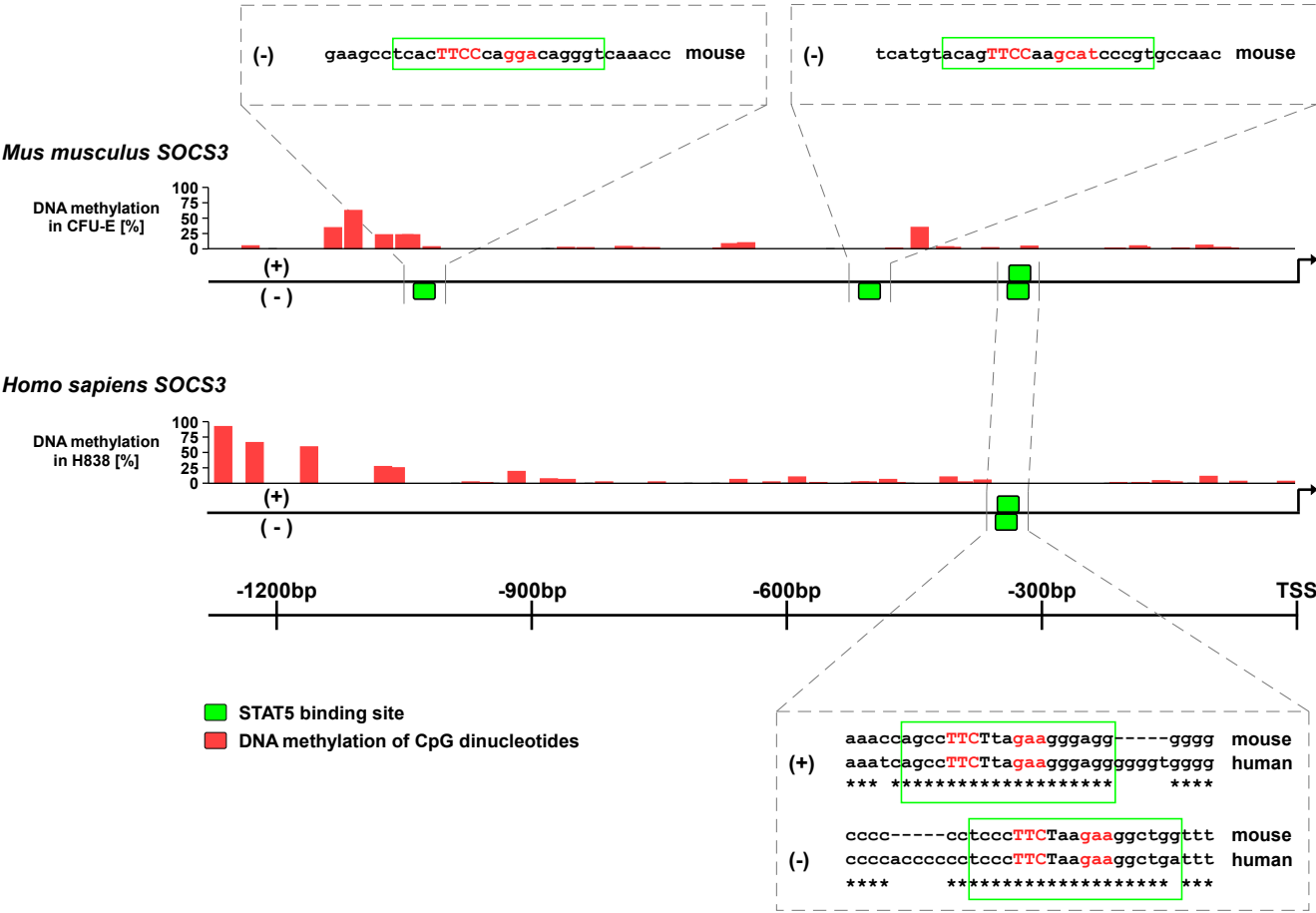

Supplement: S11 Fig — (A) pSTAT5 data and SOCS3 mRNA data are shown with the parsimonious model trajectories in CFU-E cells. Shading represents estimated experimental error. The SOCS3 promoter activity in the relevant range of npSTAT5 was calculated. (B) pSTAT5 data and SOCS3 mRNA data are shown with the parsimonious model trajectory in H838 & H838-HA-hEPOR cells. Shading represents estimated experimental error. SOCS3 promoter activity in the relevant range of npSTAT5 was calculated. (C) The SOCS3 promoter was compared between Mus musculus and Homo sapiens. TSS: transcription start site of SOCS3. Strand direction is indicated relative to the SOCS3 coding sequence. Asterisks indicate conserved bases between mouse and human. The transcription factor binding site matches are displayed as green boxes within the alignment. Bases in capital letters denote the core sequence used and red bases indicate that the matrix exhibits a high conservation at this position. Methylation of CpG islands measured by MassARRAY in CFU-E and H838 cells is indicated. (PDF) [file pcbi.1005049.s013.pdf]

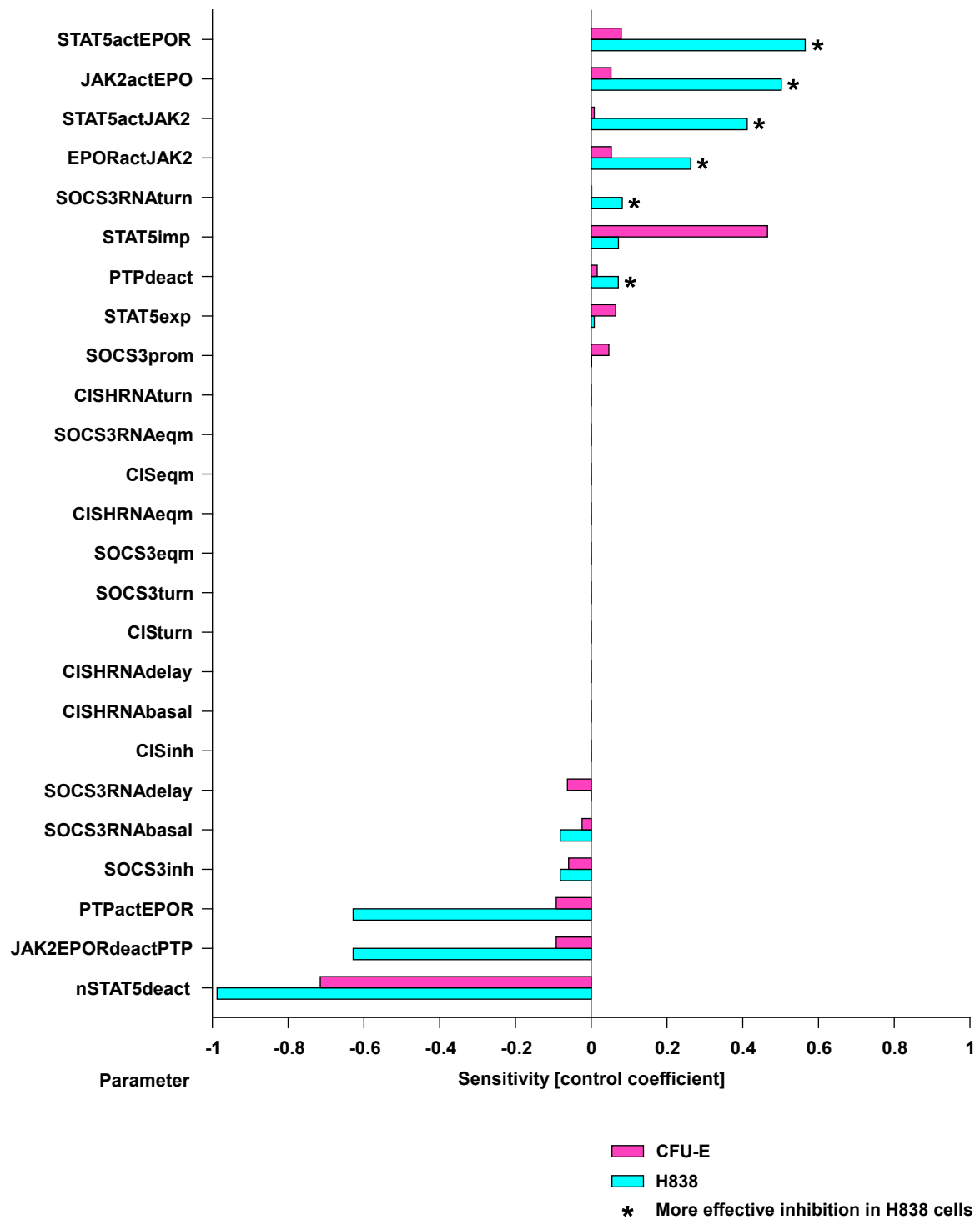

Merkle, Steiert et al., S12 Figure

Supplement: S12 Fig — Control coefficients determined for the 25 kinetic parameters in CFU-E and H838 cells are shown. The area-under-curve of npSTAT5 at 60 min after stimulation was used as read-out to calculate the sensitivities. Asterisks indicate parameters exerting more control in H838 compared to CFU-E cells. (PDF) [file pcbi.1005049.s014.pdf]
